# Mechanisms by which programmed cell death mediates immune evasion and antitumour immunity in oropharyngeal cancer

**DOI:** 10.3389/fcell.2026.1966827

**Published:** 2026-10-20

**Authors:** Songxiao Yang, Ping Zhou

**Affiliations:** Department of Radiation Oncology, Key Laboratory of Emergency and Trauma of Ministry of Education, The First Affiliated Hospital of Hainan Medical University, The First Clinical College, Hainan Medical University, Haikou, Hainan, China

**Keywords:** immune evasion, immunotherapy, oropharyngeal cancer, programmed cell death, time

## Abstract

The development and treatment response of oropharyngeal squamous cell carcinoma (OPSCC) are jointly shaped by tumour cell-intrinsic features and the tumour immune microenvironment (TIME). Programmed cell death (PCD) determines tumour cell fate and modulates antitumour immunity by altering immunogenicity, the release of immunostimulatory signals and immune-cell function. Dysregulated PCD can enable tumour cells to evade immune recognition, establish an immunosuppressive state and alter the response to immunotherapy. Here, the roles of PCD in regulating immunity in oropharyngeal cancer (OPC) are reviewed, with emphasis on understanding at the molecular level how processes involved in apoptosis, ferroptosis, pyroptosis, and autophagy contribute to either immune evasion or antitumor immunity, with consideration given to emerging pathways involving necroptosis and cuproptosis. The immunological effects of cell death are thought to be context-dependent rather than predetermined by the mode of execution. Immunologically silent death can dampen these processes, reducing the immunogenicity of tumors, impairing antigen processing and presentation, and limiting T cell recognition; conversely, immunogenic cell death (ICD) can drive functional immunological responses through the release of damage-associated molecular patterns (DAMPs), improved antigen presentation, as well as adaptive immunity activation. Next, differences between human papillomavirus (HPV)-positive and HPV-negative OPSCC are discussed regarding PCD, TIME remodeling, immune checkpoint activation, and treatment resistance, as well as their translational relevance to precision immunotherapy, radiosensitization, and chemosensitization via subgroup-based combination therapy. Given the limited OPSCC-specific evidence for several emerging PCD pathways, related findings from head and neck squamous cell carcinoma (HNSCC) studies are discussed to provide mechanistic context. Together, these insights provide a framework for understanding immune evasion in OPC and for developing PCD-targeted therapeutic strategies.

## Graphical Abstract

**Figure:**
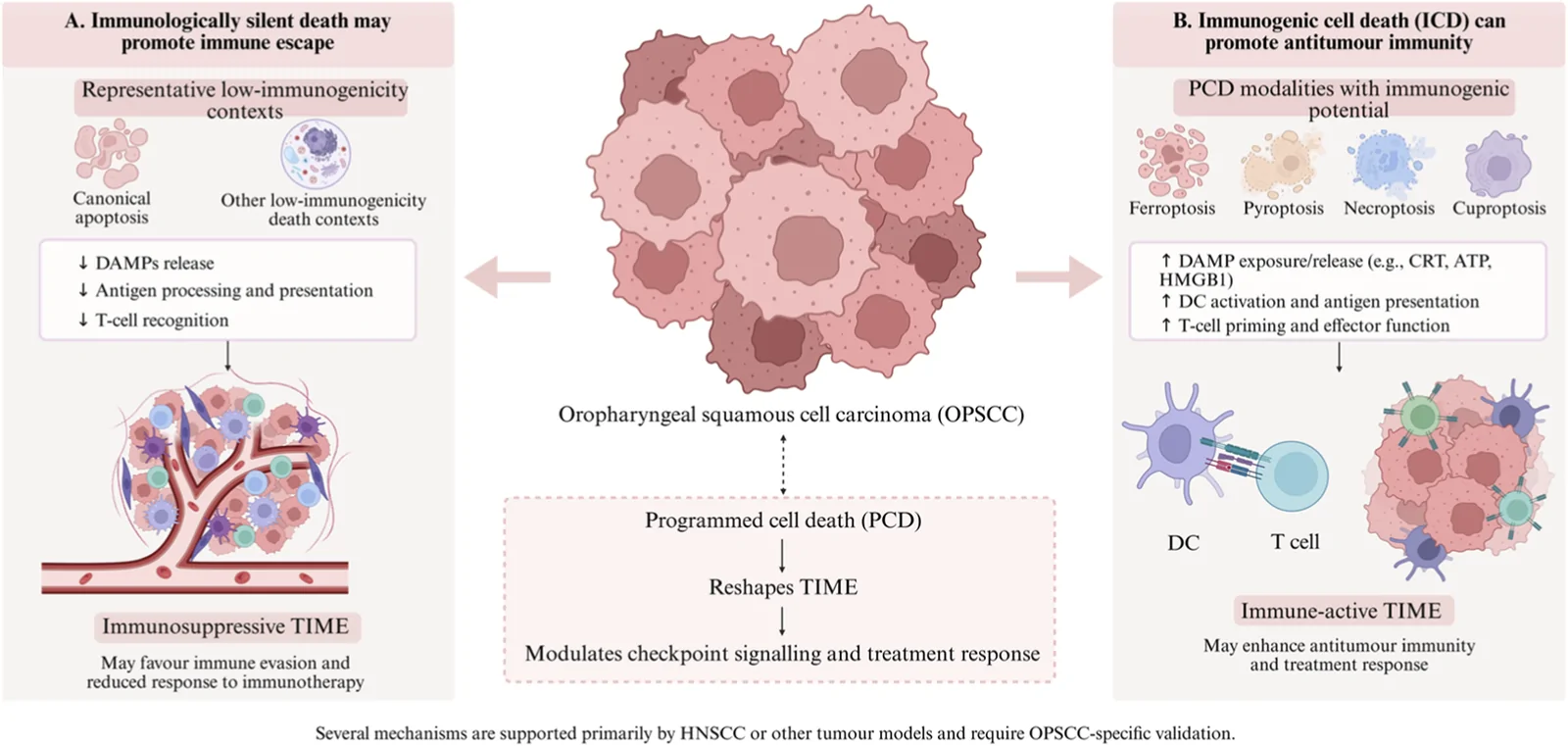

## 1 Introduction

OPSCC is a common head and neck malignancy whose epidemiology has changed markedly in recent years owing to HPV infection. Despite continuing advances in therapies such as immune checkpoint inhibitors (ICIs), some patients do not achieve durable clinical benefit because of heterogeneity in the TIME, immune evasion and treatment resistance (Liu X. et al., 2022; Noji et al., 2022). Immune-cell composition, immune-checkpoint signalling and tumour-antigen recognition within the TIME collectively shape treatment response (Tosi et al., 2022). PCD, as a key link between tumour-cell fate and immune regulation, can affect treatment outcome by altering the immunological consequences of cell death. Contemporary cell-death nomenclature defines regulated cell death (RCD) as cell death mediated by dedicated molecular machinery that can be modulated through pharmacological or genetic mechanisms, while PCD more specifically describes physiological instances of RCD (Galluzzi et al., 2018). In this Review, the operational term PCD is retained for consistency with the nomenclature used throughout the manuscript.

PCD regulates tumour cell fate, but distinct PCD modes also produce different immune effects and thereby shape the TIME and the response to immunotherapy (Wang X. et al., 2021). These immunological effects are context dependent and not only dictated by the pathway of execution. Tumor cell death is a mechanism for more than simply clearing target cells: by regulating DAMP release, immune cell recruitment and antitumor immune recognition, it can modulate tumor immunogenicity and therapeutic response. Capable of restraining adaptive antitumor immunity, some non-immunogenic types of tumor cell death can inhibit the maturation of dendritic cells (DCs) and antigen cross-presentation (Wiernicki et al., 2022). In contrast, ICD is viewed here not as a mere effector pathway but rather as an immediate immunological endpoint involving DAMP release and DC activation, migration and antigen presentation leading to enhanced T cell-induced antitumor immunity (Moriya et al., 2022; Moriya et al., 2021). Other regulated cell-death subroutines, including ferroptosis, pyroptosis, necroptosis and cuproptosis, can suppress or activate tumour immunity.

In OPC, dysregulated PCD can alter tumour–immune interactions and remodel the immune state. HPV-related OPSCC has distinctive virus-associated biology, with complex relationships among its immune features, PCD programmes and TIME state that may influence prognosis and responses to immunotherapy-related strategies (Lenoci et al., 2025; Snietura et al., 2020). Integrating PCD features with the virus-associated immune context and changes in the TIME should therefore help to define how immune evasion relates to treatment response in OPSCC.

Therapeutic strategies targeting PCD regulatory networks have attracted increasing interest. Inducing immunogenic modes of death can increase tumour immunogenicity, activate immune cells and strengthen the antitumour effects of immune-checkpoint blockade (Jeong et al., 2022). Targeting PCD pathways such as ferroptosis may instead alter tumour metabolism and susceptibility to cell death, enhance tumour clearance by radiotherapy and chemotherapy, and further modulate antitumour immunity (Lei et al., 2021). Patient stratification by HPV status, molecular subtype and immune biomarkers could refine these combinations. However, how distinct PCD modes cooperate to regulate immune evasion, antitumour immune activation and treatment response in OPC remains poorly resolved, as does the mechanistic link between PCD-dependent immune regulation and clinical benefit.

This Review encapsulates signaling mechanisms of different PCD modes that regulate immunity in OPC. This Review will elucidate immune evasion caused by immunologically silent death, how ICD activates antitumor immunity and the potential for PCD modulation in translational immunotherapy, radiotherapy and chemotherapy sensitization (Grottker et al., 2023; Chung et al., 2023). The studies evaluated here in this Review also discuss how HPV status might guide precision treatment of OPSCC. Since OPSCC is not only an anatomical subtype of HNSCC but also has squamous epithelial biology and immune-regulatory signatures in common with other HNSCC entities, mechanistic evidence derived from HNSCC is integrated when data specific to OPSCC remain limited, while site-specific and HPV-dependent differences are explicitly distinguished. Articles relevant to the subject were extracted by a focused search of PubMed and Google Scholar from 2016 to 11 September 2026; OPSCC-specific evidence was prioritized, with studies on HNSCC, oral squamous cell carcinoma (OSCC), or other tumor-derived content included if direct OPSCC evidence was sparse. This Review integrates PCD biology with virus-mediated immune characteristics and dynamic changes in the TIME with the goal of elucidating mechanisms of immune evasion by OPC, providing a rationale for combination therapeutics based on PCD as well as precision immunological approaches.

## 2 The immune microenvironment of OPC

### 2.1 Infiltration by immunosuppressive cells and immune dysregulation

The development of OPSCC is jointly governed by tumour cell-intrinsic alterations and the TIME. In HPV-related OPC, viral antigens can elicit local immune activation, while HPV-positive tumours can simultaneously display immune-checkpoint activation and exhausted T-cell states, allowing immune activation and immune dysfunction to coexist (Tosi et al., 2022; Mcinnis et al., 2023).

Tumour-associated macrophages (TAMs) are a major immunoregulatory population in the OPSCC TIME, and their polarization state is central to immune homeostasis and immune escape. Spatial multiplex analysis has shown that, in HPV-positive primary tumours and metastases, CD8^+^ T cells are located closer to tumour cells, CD163^+^ macrophages and FoxP3^+^ cells, consistent with a complex interaction network among immune-cell subsets (Tosi et al., 2022). Spatial analyses of the HNSCC immune microenvironment have identified populations of macrophages that express CD163, but CD163 expression alone should not be viewed as defining a specific functional macrophage phenotype (Blise et al., 2022). Macrophages occupy a continuum of context-dependent functional states. M1-like and M2-like descriptors are heuristic; immunosuppressive TAM states can express IL-10, TGF-β and other mediators that support tissue remodelling, angiogenesis and immune evasion. An exploratory spatial transcriptomic study identified a Th2-rich peritumoral immune signature associated with CD204+ (M2-like) macrophages, suggesting a link between Th2 polarization and macrophage state in HPV-positive OPC (Wakisaka et al., 2025). Macrophage migration inhibitory factor (MIF) can likewise promote immunosuppressive and pro-tumourigenic macrophage polarization in HNSCC (Li et al., 2022a). Notably, HPV status can shape macrophage composition and function: single-cell analysis showed reduced macrophage infiltration but stronger phagocytic activity in HPV-positive HNSCC, together with HPV-associated differences in macrophage differentiation states (Jiang Y. et al., 2022).

Myeloid-derived suppressor cells (MDSCs) and regulatory T cells (Tregs) form a core immunosuppressive network in OPC. MDSCs, including monocytic MDSCs (M-MDSCs), are important immunoregulatory myeloid populations in OPSCC. Their abundance and distribution differ between primary tumours and lymph node metastases and vary according to HPV/p16 status (Graessle et al., 2026). Tregs express high levels of FoxP3, cytotoxic T-lymphocyte-associated protein 4 (CTLA-4) and CD25 and release immunosuppressive cytokines that reduce local IL-2 availability, thereby restricting effector T cell expansion and maintaining immune tolerance (Guo et al., 2025). Stromal enrichment of Tregs and exhausted CD8^+^ T cells expressing PCD protein 1 (PD-1) and/or lymphocyte activation gene 3 (LAG-3) are prominent immune phenotypes in the OPSCC TIME and further indicate local immunosuppression (Kirchner et al., 2024). HPV infection also influences the composition and functional state of T cell subsets. Compared with HPV-positive HNSCC cohorts and with normal or inflamed tonsils, HPV-negative HNSCC contains a substantially higher proportion of Tregs without a significant change in CD8^+^ T cell frequency, suggesting that viral status shapes distinct TIME states by selectively altering immune-cell composition (Ruffin et al., 2021).

Beyond the accumulation of immunosuppressive cells, altered effector-cell function contributes to immune dysregulation in OPC. Prolonged exposure to tumour antigens can reprogramme effector T cells and constrain their antitumour activity. Natural killer cells (NK cells) and related innate immune subsets are likewise regulated by the TIME and exhibit marked functional heterogeneity. The ieILC1 subset has strong cytolytic capacity, whereas the NK-2 subset shows a transcriptional programme associated with dysfunction, implicating innate effector cells in shaping the tumour immune state (Moreno-Nieves et al., 2021). PCD links tumour cell fate to immune regulation by controlling antigen release from dying cells, inflammatory signalling and immune-cell recruitment, thereby shifting the TIME towards immune activation or immune escape. ICD promotes tumour-antigen exposure and immune activation, whereas immunologically silent death can diminish tumour recognition and sustain immunosuppression.

The OPSCC TIME is a dynamic network affected by HPV infection, immune cell interactions and tumor-derived signals and not just a stable immunosuppressive status. TAMs, MDSCs, Tregs and other suppressive populations play a role in remodeling local immune balance and limiting effector function, ultimately resulting in tumor immune evasion. But these immune landscapes can also crosstalk with molecular states related to PCD (Tosi et al., 2022; Chung et al., 2023; Graessle et al., 2026). This will require defining how each of these distinct PCD modes affects immune cell composition and remodels the TIME, which in turn will allow resolution of the heterogeneity of immune escape in OPC.

### 2.2 Functional exhaustion of effector immune cells

In the immune microenvironment of OPC, effector-cell dysfunction and the accumulation of immunosuppressive cells jointly weaken antitumour immunity. CD8^+^ T cells and NK cells eliminate tumours by recognizing tumour antigens and releasing cytotoxic molecules. Persistent antigenic stimulation, immune-checkpoint activation and local regulatory signals can, however, promote exhausted CD8^+^ T-cell states characterized by increased inhibitory-receptor expression and heterogeneous effector function, thereby constraining durable antitumour immunity (Qiu et al., 2023; Rao et al., 2024).

CD8^+^ T cell exhaustion is one of the major features of immune dysfunction in OPC. Persistent antigenic stimulation progressively shifts CD8^+^ T cells from an activated to a functionally constrained state, with sustained expression of inhibitory receptors including PD-1, T-cell immunoglobulin and mucin domain-containing protein 3 (TIM-3), and LAG-3. Stromal Tregs and intraepithelial exhausted CD8^+^ T cells expressing PD-1 and/or LAG-3 constitute major immune phenotypes in OPC, consistent with pronounced immunosuppression in the TIME (Kirchner et al., 2024). Cytotoxic tissue-resident memory T cells (Trm) are also exhausted in this setting and upregulate co-inhibitory checkpoint molecules, including HAVCR2, T cell immunoreceptor with Ig and ITIM domains (TIGIT) and LAG-3 (Cha et al., 2024). Trm subsets are functionally heterogeneous: immune-checkpoint gene-expression scores increase from TRM1 to TRM4, with TRM4 showing the most pronounced exhaustion programme (Jiang et al., 2025).

Sustained immune-checkpoint activation maintains the exhausted phenotype of effector immune cells. Increased programmed death-ligand 1 (PD-L1) expression by tumour and immune cells engages PD-1 on T cells and inhibits T cell receptor (TCR) signalling, weakening effector T cell activation and persistence. Co-expression of PD-1 with other inhibitory receptors further reinforces exhaustion. After PD-1 blockade, PDCD1 remains strongly co-expressed with immunoregulatory genes such as LAG3 and HAVCR2, suggesting that blockade of a single checkpoint is insufficient to fully restore exhausted T cell function (Rao et al., 2024). Combined checkpoint blockade across immune-cell subsets can partly reverse dysfunction: αPD-1 plus αCTLA-4 restores the effector function of exhausted CD4^+^ T cells, reduces Treg-mediated suppression and promotes OPSCC regression (Cheng et al., 2020). In the absence of specific functional T cell populations, however, PD-1 blockade does not reverse the severe dysfunction of CD8^+^ TEx-EEx tumour-infiltrating lymphocytes (TILs) in the TIME of non-responding patients with locally advanced HNSCC, further identifying effector-cell state as a major constraint on immunotherapy response (Oliveira et al., 2023).

HPV status further shapes the composition and function of effector immune cells in OPC. Persistent HPV-derived antigens can recruit T cells and generate high immune infiltration in some HPV-positive tumours, but chronic viral antigenic stimulation can also reprogramme immune cells, allowing activation and exhaustion to coexist. PD-1^+^CD4^+^ and PD-1^+^CD8^+^ T cells are both markedly increased in the parenchymal and stromal compartments of HPV-positive tumours, indicating checkpoint activation alongside virus-associated immunity (Pokryvkova et al., 2022). HPV-specific PD-1^+^TCF-1^+^ stem-like TILs retain proliferative capacity after cognate HPV peptide stimulation and can differentiate into cells expressing effector molecules, whereas terminally differentiated T cells have limited proliferative potential; exhausted T cell populations therefore retain differing degrees of functional plasticity (Eberhardt et al., 2021). Interactions between HPV-positive OPSCC cells and CD4^+^ T cells can also induce CXCL13 and IFN-γ release and establish an inflammatory TIME, although this activated state does not necessarily yield effective tumour clearance (Yan S. et al., 2024). NK cells and other innate effector cells are similarly regulated by the TIME, with altered cytotoxic function and transcriptional states that further shape local antitumour immunity.

Effector-cell exhaustion reflects the dynamic imbalance between antitumour immunity and immune escape in OPC. Dysfunction of CD8^+^ T cells and NK cells impairs immune surveillance, while HPV infection, checkpoint activation and local regulatory networks further diversify immune states. Immunotherapy can partly reverse exhaustion, but the complexity of the TIME continues to limit efficacy. Reciprocal regulation between effector exhaustion and PCD offers a framework for understanding immune evasion in OPC.

### 2.3 Effects of HPV infection and immune heterogeneity on the TIME

High-risk HPV infection is closely associated with the development of OPC and continuously shapes the composition and function of the TIME (Graessle et al., 2026). Compared with HPV-negative tumours, HPV-positive OPSCC has a distinct immune ecology in which persistent viral antigens recruit immune cells and elicit local immune activation.

Spatial transcriptomic and multi-omics studies reveal marked differences in immune organization between HPV-positive and HPV-negative OPCs. Distinct spatial transcriptomic profiles in HPV-positive and HPV-negative OPC indicate that HPV infection can remodel the local cellular ecosystem (Wakisaka et al., 2025). The TIME in OPSCC is heterogeneous across HPV-associated contexts. HPV-positive and HPV-negative tumours display distinct immune-cell compositions, while intratumoural HPV-DNA heterogeneity within HPV-positive OPSCC may further influence interferon-related and cytotoxic-lymphocyte features (Liu X. et al., 2022; Ahn et al., 2026). Multiple immune pathways are more active in HPV-positive tumours, alongside increased expression of genes associated with total TILs, CD8^+^ T cells, cytotoxic immune cells and exhausted CD8^+^ T cells, consistent with a more active immune-infiltrated state (Tosi et al., 2022).

HPV infection alters not only the extent of immune infiltration but also the composition and functional state of specific immune-cell subsets, thereby contributing to interpatient heterogeneity. Immune cells from HPV-positive and HPV-negative HNSCC have distinct transcriptional profiles, with particularly pronounced differences in helper CD4^+^ T cells and B cells but relatively similar proportions of CD8^+^ T cells and Tregs, suggesting selective effects of HPV on particular populations (Cillo et al., 2020). HPV-associated tumours show greater infiltration by CD8^+^ T cells and B cells but fewer lymphoid follicles (LF), macrophages and mast cells, further indicating virus-dependent remodelling of myeloid and lymphoid compartments (Chen et al., 2021). Although HPV-positive OPC generally contains abundant T lymphocytes, its immune ecology varies substantially among patients. HPV-positive tumours occupy multiple immune states and show greater niche diversity, demonstrating that HPV infection alone does not impose a fixed immune phenotype (Kim et al., 2020; An et al., 2026).

Single-cell analyses further reveal a more active network of immune-cell interactions in HPV-positive OPC. HPV + tumours have an immune-activated microenvironment characterized by greater infiltration of cytotoxic CD8^+^ T cells, helper CD4^+^ T cells and B cells, together with increased communication among immune-cell subsets (Li et al., 2026). High immune infiltration, however, does not necessarily confer effective tumour clearance. Persistent viral antigens can induce immune-checkpoint expression and alter effector-cell states, allowing some tumours to retain immune-evasive features despite immune activation. PCD, an important determinant of tumour immunogenicity, may further remodel the HPV-associated TIME by altering tumour-antigen release, inflammatory signalling and immune-cell recruitment.

Distinctive immune phenotypes are formed in HPV infection-associated OPC via reprogramming of immune cell composition, spatial organization and functional state. The presence of immune activation and regulatory restraint together in HPV-positive tumors suggests that a single measure cannot capture the full spectrum of their immune phenotype. The viral context, immune heterogeneity and PCD programs do not operate in isolation, but together they contribute to the complexity of immune escape and treatment response (Tosi et al., 2022; Chung et al., 2023; Sievers et al., 2026). Together, these elements shape a complex immune microenvironment in OPC characterized by both immune activation and immune evasion. Figure 1 depicts some important features of the heterogeneity of the immune microenvironment and PCD-linked immune regulation in OPSCC.

**FIGURE 1:**
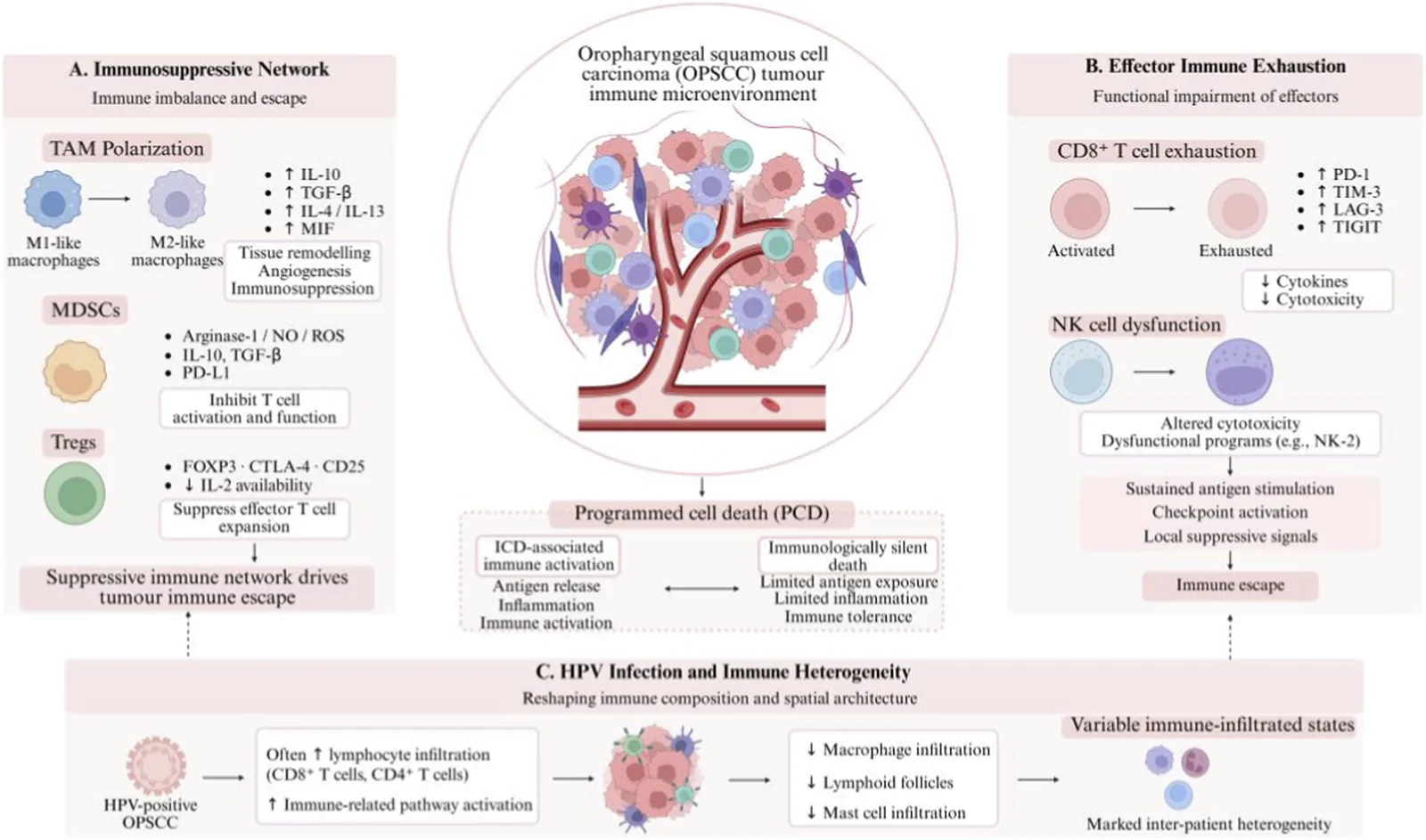
Immune microenvironmental heterogeneity and PCD-linked immune regulation in OPSCC. This schematic illustrates the major immune-microenvironmental features of OPSCC and their relationship with PCD. **(A)** The immunosuppressive network comprises distinct states of tumor-associated macrophages (TAMs), MDSCs and Tregs, and the release of suppressive mediators that inhibit T cell activation and promote immune escape. **(B)** Effector immune exhaustion: When dysfunctional CD8 T cells and NK cells are generated, there is increased checkpoint expression, reduced cytokine production and diminished cytotoxicity. **(C)** HPV-positive OPSCC is characterized by increased infiltration of lymphocytes and the activation of immune-related pathways, while checkpoint activation and pronounced interpatient heterogeneity shape immune-infiltrated states in diverse ways. Collectively, these processes constitute a dynamic TIME in which ICD-associated immune activation may augment antitumor immunity while immunologically silent death may diminish immune recognition and promote immune evasion.

## 3 Major forms of PCD and their immunoregulatory effects

### 3.1 Apoptosis: context-dependent immunological consequences and antigen escape

Apoptosis is a major form of PCD that maintains tissue homeostasis through caspase-dependent cell clearance (Liu et al., 2024). Canonical apoptosis is generally considered relatively non-inflammatory; however, its immunological consequences are context dependent, and treatment-induced apoptotic death can coexist with ICD-associated mediator release and antitumour immune activation. In tumours, dysregulated apoptosis enables malignant cells to escape both death and immune clearance, while the low immune visibility of apoptotic cells further favours immune evasion.

Within the spectrum of HNSCC, including OPSCC as a major anatomical subtype, evasion of apoptosis is a major mechanism sustaining tumour survival and treatment resistance. These HNSCC-derived findings provide mechanistic insights into apoptotic regulation in OPSCC, although tumour-site-specific validation remains necessary. As a defining form of resistance to cell death, it allows HNSCC cells to withstand intrinsic damage and therapy-induced stress (Li et al., 2024; Ow et al., 2020). Because OPSCC is an important HNSCC subtype, comparable apoptotic dysregulation may contribute to its progression and treatment response. Tumour cells can reduce sensitivity to genotoxic and therapeutic stress by strengthening anti-apoptotic signals, suppressing pro-apoptotic pathways and rewiring mitochondrial death networks. Aberrant BCL-2 family protein expression can inhibit BAX- and BAK-mediated mitochondrial outer-membrane permeabilization, reduce cytochrome c release and block the caspase-9/caspase-3 cascade, thereby promoting tumour-cell survival, invasion and migration (Liu et al., 2024; Becker et al., 2025). Conversely, strengthening pro-apoptotic signalling can restore susceptibility to death. Eugenol induces apoptosis-like death in HNSCC cells, marked by increased Bax and Bad, reduced Bcl-2, and greater caspase-3/7, caspase-8 and caspase-9 activity (Racea et al., 2023). Increasing Bax and PAR-4 while suppressing Bcl-xL and Bcl-2 can likewise activate caspase-dependent apoptosis and reduce VEGF, endothelin-1 and CTGF expression, thereby limiting tumour-associated angiogenesis and progression (Haggagy et al., 2024).

Dysregulated apoptosis affects not only tumour-cell survival but also antigen presentation and immune recognition by altering the immunogenicity of dying cells. Because membrane integrity is largely preserved during canonical apoptosis, exposure or release of DAMPs, including adenosine triphosphate (ATP), high mobility group box 1 (HMGB1) and calreticulin (CRT), are limited. Dying cells consequently fail to fully activate antigen-presenting cells (APCs), restricting the maturation of DCs and subsequent T cell priming. Compared with ICD, immunologically silent apoptosis provides insufficient stimulatory signals, reducing tumour-antigen exposure and immune recognition and promoting tolerance. In HNSCC, altered regulators of apoptosis can further modify tumour immunity; for example, SMYD3 depletion induces a type I interferon (IFN-I) response and increases expression of antigen-presentation genes, implicating apoptotic networks in tumour immune visibility (Nigam et al., 2023). HPV status might therefore further affect treatment-associated apoptotic responses. In a metformin clinical trial, the enhancement in tumor cell apoptosis was markedly greater in HPV-negative HNSCC than in HPV-positive OPSCC, whereas nodal specimens from patients who received metformin demonstrated increased CD8^+^ effector T-cell and FoxP3+ regulatory T-cell infiltration (Curry et al., 2018).

Dysregulated apoptotic pathways also sustain tumour stemness and alter tumour-cell clearance under therapeutic pressure. An anti-apoptotic state reduces therapy-induced death and may weaken immune-mediated tumour clearance. A CD276 antibody, for example, eliminates HNSCC tumour stem cells in a CD8^+^ T cell-dependent manner and activates caspase-3 in BMI1^+^ tumour stem cells, suggesting that reactivating apoptosis can strengthen immune-mediated clearance (Wang C. et al., 2021). AARS1 silencing also suppresses HNSCC-cell proliferation, migration and resistance to apoptosis (Chen S. et al., 2025), whereas CREB5 silencing activates mitochondrial apoptosis and reverses cisplatin resistance, an effect attenuated by TOP1MT overexpression (Tong et al., 2022). Apoptotic dysregulation therefore governs both the response to therapy and the balance between tumour-cell survival and clearance under immune pressure.

In OPC, apoptosis may contribute to immune escape by determining the immune visibility of cell death as well as tumour-cell survival. Enhanced resistance to apoptosis reduces death signals and the efficiency of antigen presentation. This link between apoptotic dysregulation and immune evasion is jointly shaped by tumour-intrinsic death networks, the immune microenvironment and antigen-presentation state.

### 3.2 Pyroptosis: inflammatory amplification and context-dependent immune activation

Pyroptosis is an inflammatory form of PCD mediated by gasdermin family proteins. Membrane-pore formation and the release of inflammatory mediators and chemokines promote immune activation and remodel the TIME (Wang S. et al., 2023). Unlike immunologically silent apoptosis, pyroptosis can amplify inflammatory signals to regulate immune-cell recruitment and antitumour responses.

Pyroptosis requires activation of gasdermin family effectors, including GSDMA, GSDMB, GSDMC, gasdermin D (GSDMD), gasdermin E (GSDME) and DFNB59. The N-terminal fragment generated by GSDMD cleavage perforates the plasma membrane, causing cell swelling and rupture and promoting inflammatory-factor release (Shen et al., 2021; Li et al., 2023). Depending on the upstream trigger, pyroptosis proceeds mainly through the canonical caspase-1-dependent inflammasome pathway or the non-canonical caspase-4/5/11 pathway. Inflammasome activation drives the maturation and release of IL-1β and IL-18, amplifying immune signals and altering immune-cell recruitment (Zhou et al., 2022). In HNSCC, pyroptosis-related gene-expression signatures are associated with distinct immune features and clinical outcomes, implicating pyroptosis-related molecular patterns in the tumour immune ecosystem (Zhou W. et al., 2024).

Amplification of inflammatory signals by pyroptosis can further alter the composition of the TIME and the antitumour response. Pyroptosis-associated expression patterns are closely linked to the immune infiltrate. In HPV-positive HNSCC, subgroups with high pyroptosis-related signatures are associated with greater infiltration of memory B cells, CD8^+^ T cells, activated CD4^+^ memory T cells, activated NK lymphocytes and M1-like macrophages, consistent with a more immune-infiltrated status (Li et al., 2023). Pyroptosis-related molecular patterns are also correlated with immune-inflamed, immune-excluded and immune-desert phenotypes, indicating an association between pyroptosis-related signatures and TIME architecture (Long et al., 2023). These associations do not directly show the level of active gasdermin-dependent cell death through pyroptosis. Yet pyroptosis-induced inflammation does not invariably produce effective tumour clearance; its outcome depends on immune-cell function, checkpoint expression and local regulatory networks. Some inflammatory HNSCC subgroups concurrently upregulate checkpoint genes, underscoring the context dependence of pyroptosis-associated immune activation (Nan et al., 2023). However, direct comparative evidence for gasdermin-dependent pyroptosis between HPV-positive and HPV-negative OPSCC remains limited.

Pyroptosis is closely linked to immunotherapy response and can regulate antitumour immunity. By increasing tumour immunogenicity, recruiting immune cells and activating effector responses, pyroptosis-related molecular features are associated with immune status and potential responses to immunotherapy (Lu L. et al., 2022; Liu D. et al., 2023). Direct induction of tumour-cell pyroptosis has therefore emerged as a potential therapeutic strategy (Cai et al., 2021). CTLA-4 blockade activates CD8^+^ T cells and increases IFN-γ and TNF-α secretion in the TIME; these cytokines engage the STAT1/interferon regulatory factor 1 (IRF1) axis, induce tumour-cell pyroptosis and stimulate further release of inflammatory factors and chemokines, creating a positive-feedback loop that amplifies immunity (Wang S. et al., 2023). Thus, pyroptosis is not only a form of tumour-cell death but may also serve as an important regulatory mechanism linking immunotherapeutic stimulation to amplification of antitumour immunity.

In OPC, pyroptosis may shape antitumour immunity by regulating inflammatory signals, immune-cell recruitment and TIME remodelling. Its activation can increase tumour immune visibility and promote immune responses, but the outcome depends on tumour state, microenvironmental features and treatment context; excessive inflammation may instead disrupt immune homeostasis. Interactions between pyroptosis and other PCD modes jointly shape immune escape and immune activation.

### 3.3 Ferroptosis: immune regulation through lipid peroxidation

Ferroptosis is a form of PCD driven by iron-dependent lipid peroxidation. It is characterized by the accumulation of lipid reactive oxygen species (ROS) and oxidative damage to membrane lipids and is regulated differently from other death modes (Liu F. et al., 2022; Jiang W. et al., 2021). Ferroptosis is now recognized not only as a determinant of tumour-cell survival but also as a regulator of antitumour immunity through its effects on the TIME.

Because OPSCC is a major subtype within the HNSCC spectrum, most mechanistic evidence on ferroptosis comes from HNSCC models. These findings provide a mechanistic basis for investigating ferroptotic dysregulation in OPSCC, although OPSCC-specific experimental validation remains limited. Resistance to ferroptosis enables HNSCC cells to adapt to oxidative stress and maintain malignant phenotypes. Tumour cells limit lethal lipid peroxidation by strengthening antioxidant defences, maintaining lipid homeostasis and rewiring iron metabolism. Glutathione peroxidase 4 (GPX4), a central ferroptosis regulator, clears lipid peroxides, limits lipid ROS accumulation, maintains redox homeostasis and suppresses ferroptosis (Yu et al., 2022). The cystine/glutamate antiporter (xCT), encoded by solute carrier family 7 member 11 (SLC7A11), supports glutathione (GSH) synthesis and thereby supplies the GPX4-dependent lipid-peroxide detoxification pathway. IL-6 transcriptionally activates xCT through JAK2/STAT3 signalling and reverses the ferroptosis and growth suppression caused by xCT knockdown or the ferroptosis inducer erastin, indicating that inflammatory signalling can confer ferroptosis resistance by reinforcing antioxidant capacity (Li M. et al., 2022). CAV1 likewise suppresses ferroptosis and promotes HNSCC-cell proliferation, migration and invasion, further showing that escape from ferroptosis supports adaptation to oxidative stress (Lu T. et al., 2022).

Ferroptosis determines sensitivity to oxidative damage and can also remodel the TIME and immune function through lipid peroxidation-associated signals. In HNSCC, ferroptosis-related molecular features are closely linked to tumour immune state. A ferroptosis-related DNA methylation signature was also significantly correlated with immune cell composition in HNSCC (Xu Y. et al., 2022). Ferroptosis-related gene-expression signatures FER3, FER4, FER6 and FER12 correlated with high-risk patient groups and adverse microenvironmental features comprising suppressed activity of immune components within the tumor microenvironment as well as increased stromal involvement in HPV-positive OPSCC (Lenoci et al., 2025). Although such molecular signatures provide prognostic and immune-association evidence for ferroptosis, they do not directly show active cell death by ferroptosis or that ferroptosis can cause effects in the immune microenvironment. These HNSCC data provide a mechanistic basis for examining the intersection of metabolic abnormalities, PCD and the immune microenvironment in OPSCC.

Inducing ferroptosis has the potential to strengthen antitumour immunity and improve treatment response. In HNSCC, ferroptosis-related molecular features are associated with the tumour immune microenvironment, while MTDH-mediated ferroptosis can enhance radiosensitivity (Cao et al., 2024). Lipid-peroxidation products and oxidative-damage signals generated during ferroptosis may increase tumour immune visibility, facilitate immune recognition and clearance, and weaken immune-evasive phenotypes. Ferroptosis-inducing strategies could also improve effector-cell function by remodelling the TIME and enhance sensitivity to immunotherapy. These effects are highly context dependent, however, and are shaped by tumour metabolism, redox homeostasis and local microenvironmental conditions. Ferroptosis-associated redox imbalance can itself alter immune-cell composition and the TIME (Zhao et al., 2023). Therapeutic induction of ferroptosis will therefore require tumour-specific control.

Ferroptosis may influence immune regulation in OPC beyond its effects on tumour-cell death. Lipid peroxidation-driven oxidative stress affects tumour survival, the immune microenvironment and treatment response. Resistance to ferroptosis can favour immune escape, whereas its induction may strengthen antitumour immunity. These immune effects are strongly context dependent, and available evidence suggests HPV-associated differences in ferroptosis-related immune patterns; however, direct mechanistic comparisons between HPV-positive and HPV-negative OPSCC remain limited.

### 3.4 Autophagy regulation and autophagy-dependent cell death (ACD): cell fate and immune homeostasis

Autophagy is a regulated degradative process with cytoprotective, metabolic and immunoregulatory functions. ACD refers specifically to cell death that mechanistically requires the autophagic machinery; autophagy accompanying cell death alone does not establish ACD (Denton and Kumar, 2019). Dysregulated autophagy can also regulate immune signals and the TIME, thereby contributing to immune escape or antitumour immunity. Evidence from HNSCC provides important mechanistic insights into autophagy-mediated regulation in OPC, particularly because OPSCC-specific studies remain relatively limited.

Aberrant autophagy can sustain malignant phenotypes and enhance adaptation in HNSCC. The ANXA6/TRPV2 axis promotes HNSCC-cell migration by activating autophagy and contributes to lymph node metastasis (LM) (Wang M. et al., 2023). Increased autophagy also helps to maintain HNSCC-cell stemness, potentially by promoting GSK3β degradation, increasing nuclear translocation of β-catenin and activating downstream transcriptional programmes (Liu Y. et al., 2023). These findings primarily describe tumour-promoting or cytoprotective functions of autophagy rather than *bona fide* ACD. Autophagy is therefore not simply a degradative process; it regulates signalling, stemness and metabolic adaptation to shape tumour-cell fate. Its effects are dual. In OPSCC and related oral/oropharyngeal SCC cohorts, LC3B expression and autophagy-related long non-coding RNA (lncRNA) signatures have been associated with clinical outcome (Lai et al., 2018; Jiang Q. et al., 2021). However, these observational and computational associations do not establish ACD.

Dysregulated autophagy can also promote immune evasion in HNSCC by modulating immune pathways. In the context of HPV infection, viral factors can exploit host autophagy to weaken antitumour surveillance. HPV16 E7 promotes autophagy-dependent degradation of stimulator of interferon genes (STING) and, through NLRX1, strengthens autophagic suppression of STING/IFN-I signalling, thereby reducing IFN-I-mediated immune activation (Kandathil et al., 2024). The mechanism serves as a model for autophagy-mediated immune regulation and not a proof of concept for ACD. Importantly, single-cell and multiplex immunofluorescence analyses revealed that ATG7 was correlated with high PD-L1 expression in myeloid cells through a noncanonical autophagy-independent function without impacting PD-L1 expression on tumor cells (Guo et al., 2022). Consequently, this finding represents an ATG7-dependent modulation of immunity rather than evidence for ACD. Autophagy can thus alter the TIME by regulating immunosuppressive cell populations and checkpoint expression as well as tumour-cell biology. Given the close association between OPSCC and HPV infection, these HNSCC findings provide relevant context for understanding autophagy-mediated immune regulation in virus-associated OPC.

Autophagy interacts with other forms of PCD and can alter treatment response. In HNSCC cells, CDK4/6 inhibition with abemaciclib induced pro-survival autophagy, while combined CDK4/6 and autophagy inhibition synergistically inhibited cell viability, induced apoptosis *in vitro* and suppressed tumour growth in preclinical models (Duah et al., 2023). Long-term cetuximab exposure decreased Akt/mTOR activity and enhanced autophagy-associated alterations in HNSCC cells, correlating treatment-mediated cell-cycle arrest with autophagy induction (Okuyama et al., 2021). The biological consequences of autophagy nevertheless depend strongly on tumour type, metabolic state, treatment and the immune microenvironment. Autophagy may promote tumour-cell death and immunogenicity in one context, yet sustain tumour survival and immunosuppression in another.

There has been little direct evidence for *bona fide* ACD in OPSCC. Importantly, available OPSCC and HNSCC studies suggest that autophagy-related processes can modulate processes associated with tumor adaptation, stemness, immune-related signaling and therapeutic sensitivity, but their relationship with autophagy-dependent execution of cell death requires further validation (Kandathil et al., 2024; Chen Y. et al., 2022; Lee et al., 2026). The functional heterogeneity of autophagy-related processes as it relates to context and the interplay with HPV infection, tumor heterogeneity and other modes of PCD remains partly elucidated. In summary, various PCD modalities have coordinated roles in modulating tumor immunity via the regulation of antigen exposure, inflammatory signaling, lipid metabolism and immune cell function. Figure 2 summarizes the main mechanisms of apoptosis, pyroptosis, ferroptosis and autophagy-related responses in regulating the immune response in OPC.

**FIGURE 2:**
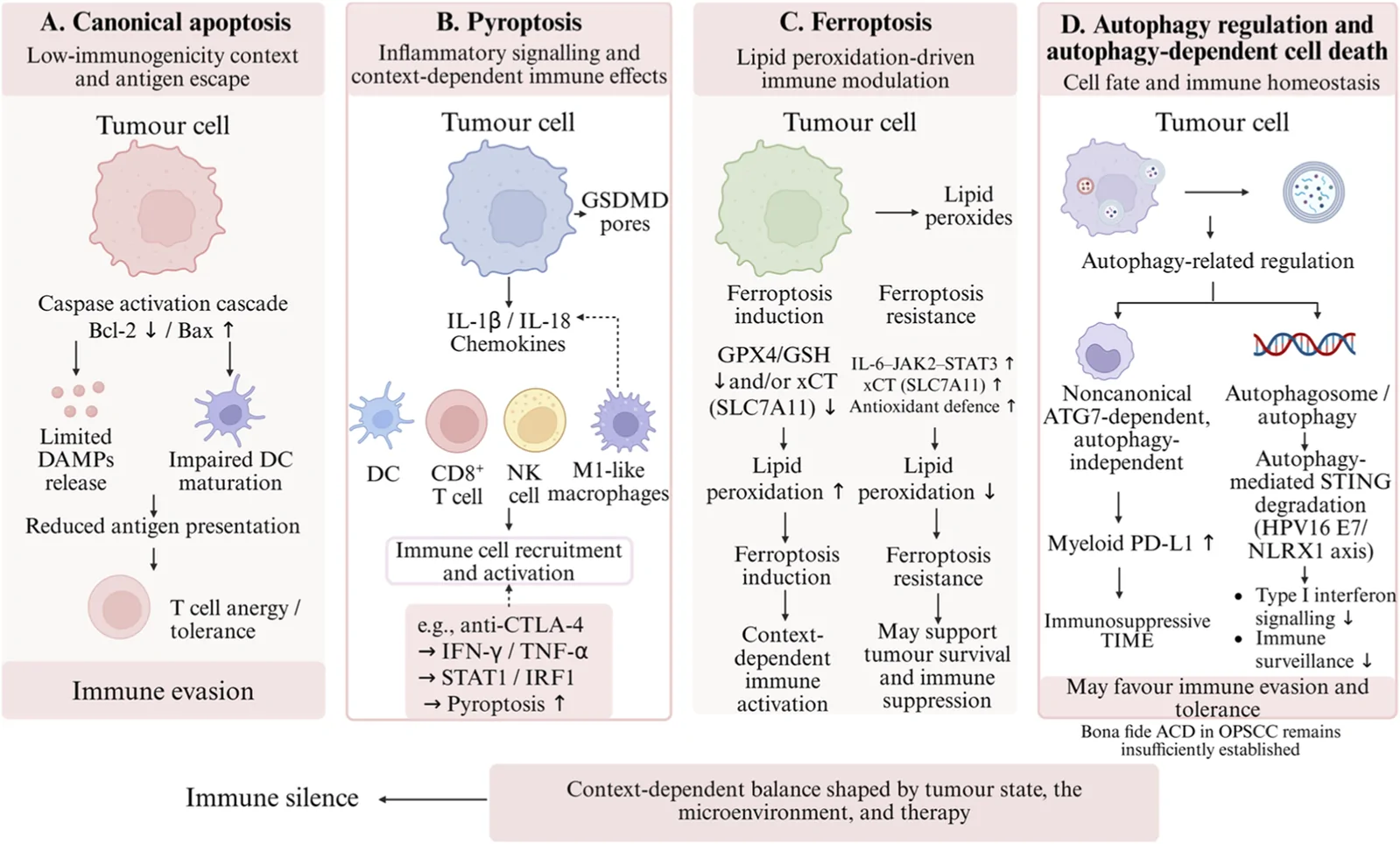
Major PCD-related pathways and their context-dependent immunoregulatory consequences relevant to OPSCC. This figure provides an overview of the key PCD pathways discussed in this manuscript and their context-dependent immunological effects associated with OPSCC. **(A)** In most cases, canonical apoptosis is associated with a low-immunogenicity context; restricted DAMP release and anti-apoptotic imbalance can inhibit DC maturation, limit antigen presentation and lead to T cell anergy or tolerance. **(B)** Pyroptosis is a gasdermin-mediated inflammatory mode of PCD characterized by membrane pore formation and IL-1β/IL-18 release. Overall immunological effects are context dependent, although the signaling pathways leading to pyroptotic cell death can facilitate immune cell recruitment and activation. **(C)** Lipid peroxidation and antioxidant defense regulate ferroptosis. Decreased GPX4/GSH and/or xCT (SLC7A11) activity favors ferroptosis induction, whereas IL-6–JAK2–STAT3-driven xCT upregulation and improved antioxidant defense promote ferroptosis resistance; their immune effects remain conditional. **(D)** Different mechanisms of autophagy-related regulation of immune homeostasis: Myeloid PD-L1 increases via an autophagy-independent, noncanonical ATG7-dependent pathway; STING is degraded through the autophagy-mediated HPV16 E7/NLRX1 axis that dampens IFN-I signaling and immune surveillance. Direct evidence for genuine ACD in OPSCC is still limited. Where direct OPSCC evidence is limited, mechanistic data derived from HNSCC or other tumors are used as context, not as direct validation for OPSCC.

## 4 Mechanisms through which PCD may promote immune evasion in OPC

### 4.1 Immunologically silent death impairs tumour-antigen presentation

The immune effects of PCD are jointly influenced by the death programme, the release of immunogenic signals and antigen presentation. If the death of tumor cells cannot elicit enough activating signals, it might provide minimal aid to DC activation and the resultant antitumor immunity, whereas defective MHC-dependent antigen presentation decreases T cell recognition and may promote immune evasion and treatment resistance (Grottker et al., 2023; Yan S. et al., 2024; Zhang et al., 2022). While direct evidence from OPSCC is limited, mechanistic studies in HNSCC provide relevant context because of their shared squamous carcinoma origin and overlapping immune-regulatory networks.

Defective antigen presentation is a major route through which immunological silence promotes tumour immune escape. Tumour cells chiefly present endogenous antigens to CD8^+^ T cells through major histocompatibility complex class I (MHC-I). Loss of MHC-I antigen-presentation capacity reduces tumour immune visibility and confers resistance to immunotherapy (Burr et al., 2019). Tumour-intrinsic nucleophosmin (NPM1) can also suppress IRF1-dependent antigen presentation, reduce tumour immunogenicity and establish an immunosuppressive TIME, thereby strengthening immune evasion (Wang X. et al., 2024). Immunologically silent states and defective antigen presentation can therefore act together to reduce tumour recognition and reconfigure tumour–immune interactions.

Unlike immunologically silent death, ICD activates antitumour immunity through the release of DAMPs. These DAMPs engage pattern recognition receptors (PRRs) on DCs, macrophages and monocytes, promoting the activation and maturation of APCs and the transport of tumour antigens to lymph nodes, where adaptive immune responses are initiated (wang X. et al., 2021; Checcoli et al., 2020). Immunologically silent death releases too few stimulatory signals to efficiently activate APCs or recruit T cells, so tumour-cell death does not progress to immune-mediated clearance. The immune signals accompanying each mode of death are therefore a major determinant of its immunological outcome.

Defective antigen presentation usually coincides with disruption of broader immune-regulatory networks, sustaining tumour immune escape. In HNSCC and related squamous cell carcinomas, cancer cells can weaken antitumour immunity by disrupting antigen presentation while strengthening immunosuppressive signalling (Zhang J. et al., 2024; Chin et al., 2025). Suppression of interferon-dependent inflammation and reduced antigen-presentation capacity can also allow dominant tumour clones to evade surveillance by lowering host immune recognition (Chin et al., 2025). Aberrant activation of checkpoint molecules further impairs T cell effector function, enabling presentation defects and suppressive signals to jointly maintain tolerance. Immunological silence therefore affects both the sensing of dying cells and the multilevel networks that support persistent immune evasion.

In OPC, insufficient immunostimulatory signals induced by tumor cell death may limit tumor immunogenicity and promote immune evasion, whereas inadequate antigen presentation can be an additional barrier to tumor recognition. Immune consequences of the death of tumor cells are highly context dependent and codetermined by the modality of death, antigen presentation status, and the local immune environment (Grottker et al., 2023; Yan S. et al., 2024). Unpacking the interplay of PCD with antigen presentation networks may reveal the regulatory hubs that maintain immune tolerance and provide a target for re-establishing tumor recognition.

### 4.2 Persistent inflammation promotes an immunosuppressive microenvironment

The release of inflammatory mediators and damage-associated signals by inflammatory PCD can reshape tumor immune ecology. In HNSCC models, pyroptosis may increase DAMP release and promote DC maturation and T-cell recruitment, whereas NLR family pyrin domain containing 3 (NLRP3)-mediated inflammatory signaling in TAMs promotes M2-like macrophage differentiation and the accumulation of immunosuppressive myeloid cells (Zhang Y. et al., 2026; Chen L. et al., 2023). These results demonstrate the potential of inflammatory signaling to promote either antitumor immunity or an immunosuppressive TIME, depending on the context, and provide mechanistic insights into OPSCC.

The inflammatory signals released during pyroptosis provide a direct link between PCD and immune-microenvironmental remodelling. Unlike immunologically silent apoptosis, pyroptosis causes cell swelling, loss of plasma-membrane integrity and release of inflammatory contents. Pores formed by gasdermin family proteins allow damage-associated molecules and inflammatory mediators to exit the cell; release of IL-1β and IL-18 further activates local inflammation and regulates immune-cell recruitment (Zhou et al., 2022; Yan B. et al., 2024). In HNSCC, pyroptosis-related gene-expression signatures have been associated with tumour-microenvironmental features, immune-cell infiltration and clinical outcomes (Zhou W. et al., 2024; Wu et al., 2022), although these associations do not directly demonstrate gasdermin-dependent pyroptotic execution.

Myeloid remodelling induced by persistent inflammation is a central mechanism of immune escape. In HNSCC models, NLRP3 expression in TAMs correlated with worse prognosis, while depletion of NLRP3 inhibited M2-like macrophage differentiation and decreased the accumulation of both TAMs and MDSCs, all supporting a role for NLRP3-dependent myeloid remodelling (Chen L. et al., 2023). In HPV-positive OPSCC, an immune-inflamed primary tumor phenotype with high CD8 and low CD163 expression was linked to improved progression-free survival (Nakkireddy et al., 2024). Conversely, a high density of CD163+ M2-like macrophages was associated with worse locoregional control, early metastasis-free survival and overall survival in HR-HPV-negative OPSCC (Snietura et al., 2020). These observations show that the immune states associated with macrophages are prognostically significant depending on context and HPV status.

Persistent inflammation can interact with other immune-evasion mechanisms to further constrain antitumour responses. Single-cell analysis has identified a complex interaction network linking MDSCs with cytotoxic CD8^+^ T cells, SPP1^+^TAMs and endothelial cells; this network may establish an immunosuppressive microenvironment and promote tumour angiogenesis (Jiang W. et al., 2023). Tumour cells can simultaneously reduce immune surveillance by upregulating negative regulators such as PD-L1 and thereby evade killing by immune effector cells (Wuerdemann et al., 2020). Inflammatory PCD is intrinsically bidirectional: moderate activation may enhance immune recognition, whereas persistent dysregulated signalling can promote tolerance. Its ultimate effect is therefore determined by the duration of inflammation, the immune-cell composition and the local microenvironment.

PCD-associated inflammatory signaling can underlie the balance between immune evasion and antitumor immunity in OPC through immunogenic signals, myeloid remodeling and remodeling of the TIME. The HNSCC studies inform mechanistic understanding of OPSCC; however, the reciprocal effects of inflammatory PCD and their contextual variation across disease need to be more clearly defined (Chung et al., 2023; Zhao et al., 2023).

### 4.3 Metabolic reprogramming cooperates with cell-death programmes to promote immune tolerance

Tumor metabolic reprogramming provides energy and biosynthetic precursors to support continuous cancer cell proliferation, and in some cases modulates immune activity by remodeling the immune microenvironment. Moreover, cancer metabolic aberrations can affect immune checkpoint expression and T cell dysfunction in HNSCC (Zhang XY. et al., 2024), while metabolic regulators may also change vulnerability to ferroptosis (Cao et al., 2024). HNSCC findings add mechanistic context for future investigation of possible crosstalk between metabolic remodeling, PCD and the TIME in OPC.

Enhanced glycolysis and lactate accumulation are hallmarks of metabolic reprogramming that drives immune evasion. In a mouse model of HPV-positive OPC, increased extracellular lactate induces PD-L1 transcription in tumour cells and promotes expression of other co-inhibitory checkpoint molecules, thereby weakening antitumour surveillance (Verma et al., 2020). CRISPR/Cas9-mediated PKM2 knockdown suppresses the Warburg effect and reduces lactate production, with concomitant decreases in HIF-1α and PD-L1 expression, indicating that glycolytic remodelling promotes immune escape by altering hypoxic signalling and checkpoint expression (Zou et al., 2025). FTO-mediated m6A demethylation also increases expression of the transcription factors c-Jun, JunB and C/EBPβ, which further drive glycolytic reprogramming and confer a metabolic advantage under immune pressure (Liu et al., 2021). Metabolic abnormalities thus support tumour-cell survival while strengthening immunosuppressive signals that establish tolerance.

Changes in metabolic state can alter susceptibility to PCD and reshape the tumour immune environment. Ferroptosis, a prototypical metabolism-associated form of PCD, is jointly controlled by lipid metabolism and antioxidant defences. MTDH promotes ferroptosis by regulating GPX4 expression and increases the radiosensitivity of HNSCC cells, showing that metabolic regulators can alter ferroptotic susceptibility (Cao et al., 2024). Conversely, increased ERO1α, a functional target downstream of mechanistic target of rapamycin complex 1 (mTORC1), upregulates SLC7A11, induces resistance to ferroptosis and promotes malignant behaviour in mTORC1-activated cells (Wang Z. et al., 2024). Ferroptotic state can in turn reshape immune ecology. In an HNSCC xenograft model, RSL3-induced ferroptosis reduced MDSCs and M2-like TAMs while increasing tumour-infiltrating CD4^+^ and CD8^+^ T cells, supporting an immunomodulatory effect of experimentally induced ferroptosis in HNSCC (Zhao et al., 2023). The dynamic balance between metabolic control and PCD switching therefore determines both the propensity for tumour-cell death and the immune-response state.

Metabolic crosstalk among PCD modes adds further complexity to tumour immune regulation. Autophagy and ferroptosis regulate one another, jointly shaping the immune microenvironment and immune function in squamous cell carcinoma (Chen L. et al., 2022). Metabolic abnormalities can alter not only the threshold for a single death pathway but also the transitions and cooperation among distinct PCD modes. Increased glycolysis, for example, promotes lactate accumulation and checkpoint expression, whereas disrupted redox homeostasis changes ferroptotic sensitivity and immune-cell infiltration. Metabolic reprogramming therefore coordinates multiple PCD programmes with immune-regulatory networks to establish a tumour ecosystem characterized by tolerance.

Metabolic reprogramming cooperating with PCD might suggest an important regulatory axis of immune escape in OPC. HNSCC studies support that abnormal metabolism alters the antitumour immune balance, leading to changes in susceptibility to cell death, checkpoint expression and TIME composition (Chung et al., 2023; Song et al., 2024). This interaction is intricate and characterized by remarkable heterogeneity between disease contexts, particularly regarding HPV status, metabolic features and immune ecology in OPSCC. In addition to controlling the survival of tumor cells, PCD regulates immune escape by sculpting antigen presentation, inflammatory responses and the metabolic and immune functions of cells. Figure 3 summarizes the main mechanisms by which PCD dysregulation may promote immune evasion and treatment resistance in OPC.

**FIGURE 3:**
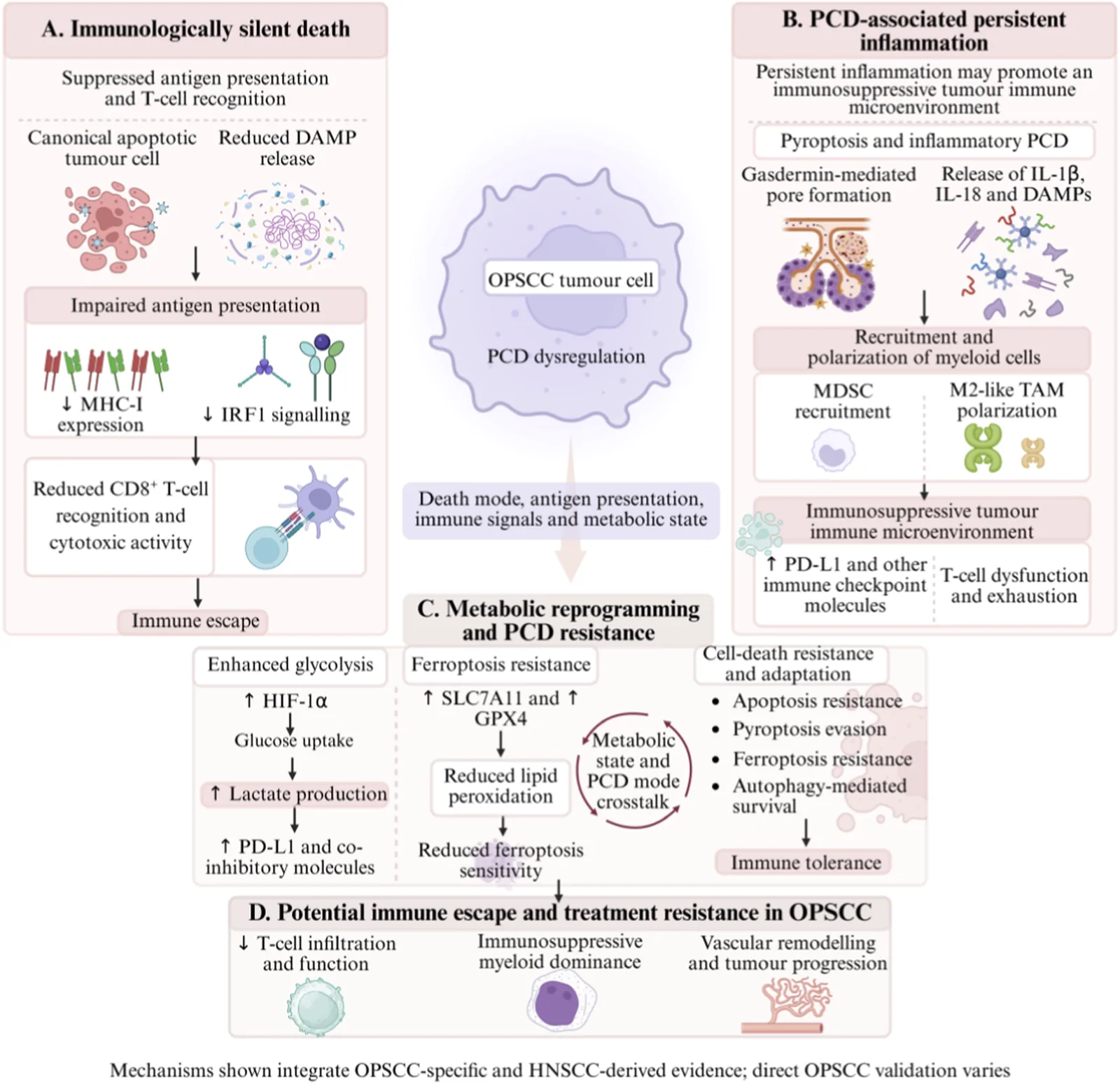
PCD dysregulation may promote immune evasion and treatment resistance in OPSCC. Schematic of possible immune escape and treatment resistance mechanisms mediated by dysregulated PCD. **(A)** Immunologically silent cell death can downregulate antigen presentation and T cell recognition by inducing lower levels of DAMPs, reducing MHC-I expression and impairing IRF1-related immune signaling to promote reduced cytotoxic effects. **(B)** PCD-induced persistent inflammation via pyroptosis-associated and other pro-inflammatory signals could result in the release of IL-1β, IL-18 and DAMPs, MDSC recruitment and M2-like macrophage polarization, possibly leading to an immunosuppressive TIME. **(C)** Metabolic reprogramming can cooperate with cell-death resistance by promoting glycolysis, lactate production, PD-L1 expression and ferroptosis resistance via SLC7A11-and GPX4-dependent mechanisms. **(D)** These convergent pathways may diminish T cell infiltration and function, augment the myeloid suppressor niche and vascular remodeling, thus ostensibly enabling immune escape and treatment resistance. Mechanisms shown are supported by OPSCC-specific and/or HNSCC-derived evidence; however, direct validation in OPSCC varies.

## 5 Mechanisms through which PCD promotes antitumour immunity

### 5.1 Release of DAMPs activates innate immunity

PCD contributes to tumour clearance and can initiate antitumour immunity by altering the immunogenicity of dying cells. ICD, a key interface between cell death and immune activation, is characterized by the exposure or release of DAMPs. These signals are sensed by the innate immune system and contribute to tumour-antigen presentation and remodelling of the immune microenvironment.

DAMP release during ICD is a key initiating event in innate immunity. Unlike immunologically silent apoptosis, immunogenic PCD releases danger signals such as ATP, HMGB1 and CRT, increasing immune recognition of dying tumour cells (Gedik et al., 2024; Sun et al., 2022). Surface-exposed CRT acts as a phagocytic signal that promotes tumour-antigen recognition and uptake by DCs; extracellular ATP can contribute to immune-cell recruitment and inflammatory signalling; and released HMGB1 engages PRRs to activate innate immune cells, linking tumour-cell death to immune surveillance (Zhao et al., 2019). DAMPs released during ICD also interact with PRRs on DCs, macrophages and monocytes, promoting the maturation of APCs and strengthening subsequent adaptive responses (Wang X. et al., 2021). Effective immune activation by PCD therefore depends not only on the death mode but also on the accompanying release of immunostimulatory DAMPs.

Non-HNSCC tumor evidence has shown that cuproptotic tumor cells could be associated with ICD signaling with DAMP release and DC activation (Lei et al., 2026), while NO was able to induce ICD and synergize with antitumor immunity in immunocompetent tumors (Jiang W. et al., 2022). Cetuximab and cisplatin together induced apoptotic cell death with ICD-associated mediators in HNSCC models, while moderate apoptosis was accompanied by an antitumor immune response (De Azevedo et al., 2022). These observations demonstrate that ICD can be associated with distinct cell-death programmes and should be considered a functional immunological outcome rather than one particular mode of execution.

DAMP-mediated innate immune activation is closely linked to immunotherapy response. The release of ICD-associated DAMPs and their interactions with innate immune receptors can initiate antitumour immunity and may have predictive value for immunotherapy in HNSCC (Zhang J. et al., 2024). DAMPs released during PCD can also enhance recognition of tumour-derived antigens and shift the TIME towards immune activation. These effects are nevertheless context dependent and vary with the death mode, DAMP composition, tumour immune context and local inflammatory environment. Excessive or sustained DAMP release may provoke pathological inflammation and disrupt immune homeostasis, underscoring the need to define its regulation and consequences across PCD modes.

The release of DAMP molecules associated with PCD can change the immunogenic properties of dying cells and connect tumor damage to immune recognition in tumors. While ICD-associated DAMPs can enhance tumor recognition and strengthen antitumor immunity, little is known of their role in OPSCC (Grottker et al., 2023; Zhao et al., 2023). Integration of DAMP profiles across PCD modes according to HPV status, tumor heterogeneity and the local immune environment should delineate mechanisms by which antitumor immunity is initiated in OPC.

### 5.2 Enhanced antigen presentation activates adaptive immunity

PCD regulates the initiation of adaptive immunity by changing the immunogenicity of dying cells as well as clearing tumour cells. ICD promotes tumour-antigen release and immune activation by APCs. In OPC and related HNSCC studies, PCD-dependent antigen release and presentation influence the establishment of adaptive responses.

Tumour-antigen release during PCD and cross-presentation by DCs are central to initiating adaptive immunity. Immunogenic death releases tumour antigens, enhances DC-dependent cross-presentation and activates tumour-specific CD8^+^ T cells, thereby strengthening antitumour responses (Yang Z. et al., 2025). Induction of ICD promotes tumour-antigen capture, DC activation and efficient antigen cross-presentation, leading to tumour-specific T cell activation and stronger adaptive immunity (Pol et al., 2020). Enhanced uptake and processing of antigens from dying cells by DCs during ICD can also generate CD4^+^ and CD8^+^ T cell responses and broaden immune effects (Zhang R. et al., 2026). Whether PCD yields effective immune activation therefore depends on its capacity to induce antigen release and presentation as well as on the death mode itself.

Distinct forms of PCD can promote antigen presentation and adaptive immune activation by inducing ICD. Immunogenic death increases tumour-antigen release, DC maturation and antigen presentation, thereby activating CD8^+^ T cells and enabling immune-dependent tumour clearance (Yang LJ. et al., 2025). In squamous cell carcinoma models, cell death induced by photodynamic therapy (PDT) promotes DC recognition of tumour-derived antigens and their subsequent presentation to cytotoxic T lymphocytes (CTLs), strengthening antitumour immunity (Li et al., 2022c). Despite their distinct execution mechanisms, these death modes may convert dying tumour cells from clearance targets into antigen sources that activate adaptive immunity.

Antigen-presentation capacity directly affects T cell-mediated clearance and immunotherapy response. During the cancer–immunity cycle, DCs, macrophages and other APCs take up and process tumour-associated antigens or neoantigens, then cross-present them to cytotoxic CD8^+^ T lymphocytes to elicit specific antitumour responses (De Azevedo et al., 2022). Tumour cells can evade recognition by reducing their antigen-presentation capacity. EZH2 inhibition or CRISPR-mediated EZH2 loss enhances antigen presentation by HNSCC cells and increases antigen-specific CD8^+^ T cell proliferation, IFN-γ production and cytotoxicity against tumour cells, suggesting that restoration of tumour-cell antigen presentation could improve immunotherapy (Zhou et al., 2020). Adaptive immune activation is therefore jointly determined by PCD-induced antigen release and the antigen-presentation state of the tumour cells.

By promoting antigen release and presentation, PCD converts a death event into a signal that initiates adaptive immunity. ICD-associated antigen presentation can drive DC maturation and T cell activation, but how different death modes regulate antigen-presentation networks in OPSCC remains unclear. Their effects need to be defined in relation to HPV status, tumour-antigen features and the local immune environment.

### 5.3 Remodelling the immune microenvironment to increase sensitivity to immunotherapy

PCD determines tumour-cell fate and can also shape antitumour immunity by releasing immunogenic signals, altering immune-cell composition and remodelling the TIME. Immunoregulatory forms of PCD can activate DCs, recruit T cells and shift an immunosuppressive TIME towards an immune-responsive state. In OPC, such remodelling may influence the response to immunotherapy.

PCD-induced immunogenic changes can reconstruct the TIME by recruiting immune cells and restoring their function. Immunogenic signals generated during PCD activate DCs and T cell-mediated antitumour immunity and can increase sensitivity to immunotherapy by altering immune-cell composition (Duurland et al., 2024). PCD-driven TIME remodelling can also restore immunostimulatory signalling, activate innate immunity and improve therapeutic efficacy (Sun et al., 2025). Pyroptosis-related molecular states are associated with differences in immune-cell infiltration and immune phenotypes and may therefore help characterize immunotherapy-relevant tumour states in HNSCC (Pan et al., 2024). Its immunoregulatory effects thus extend beyond tumour-cell killing to the local immune ecology that determines the strength of antitumour immunity and the response to treatment.

Metabolism-associated PCD can further remodel the TIME and improve treatment response by activating immunostimulatory pathways. Ferroptosis is a prototypical metabolism-dependent form of PCD that perturbs tumour-cell redox homeostasis and alters tumour–immune interactions. In HNSCC models, ferroptosis induction suppresses tumour growth and activates STING-IFN-I signalling, promoting DC recruitment and maturation, antigen presentation and antitumour immunity (Li et al., 2025). This immunogenic transition increases CD8^+^ T cell infiltration and cytotoxicity and enhances the antitumour effect of carbon ion radiotherapy (CIR). DDR1, by forming a DDR1/14-3-3/Akt complex, activates the Akt/mTORC1/SREBP1/SCD1 axis, promotes monounsaturated fatty acid (MUFA) synthesis and suppresses ferroptosis. DDR1 silencing removes MUFA-dependent resistance to ferroptosis, increases tumour immunogenicity and CD8^+^ T cell infiltration and cytotoxicity, and improves the efficacy of CIR (Hu et al., 2025). Dynamic crosstalk between metabolic state and PCD susceptibility can therefore alter treatment response through changes in the TIME.

The molecular classification of cuproptosis has been linked to certain immune features in HNSCC. One study developed a cuproptosis score derived from cuproptosis-related genes (CRGs) that was related to immune infiltration, immune checkpoint expression and predicted treatment response (Huang et al., 2022), while another study constructed a CRG risk model associated with different patterns of immune cell infiltration (Jiang X. et al., 2023). These computational relationships are suggestive of, but do not substantively validate, cuproptotic cell death or causal regulation of the immune microenvironment. Combining ICI with other therapies can also convert immunologically cold tumours into immunologically hot tumours and increase sensitivity to checkpoint blockade (Chen L. et al., 2022). PCD-induced immune activation is nevertheless context dependent and is jointly determined by the mode of death, immune-cell composition, metabolic state and local inflammation.

PCD can alter the composition and functional state of TIME to enhance tumor immunity and change responses to therapies. Ferroptosis in HNSCC studies provides direct evidence of the immunoregulatory potential of PCD (Zhao et al., 2023; Li et al., 2025), but its relevance to OPSCC is still poorly defined. This mechanistic understanding of immune regulation in OPC should be enhanced by resolving PCD-TIME interactions according to HPV status, tumor heterogeneity and local immune characteristics. A summary of the key cell-death pathways and immunological consequences and the relevant contexts in which they regulate immune escape or antitumor immunity in OPC is shown in Table 1. These results demonstrate a mechanistic link between tumor cell death and innate immune activation, DC recruitment and maturation, and TIME remodeling through ICD-associated processes. Figure 4 links the major ICD-associated pathways connecting tumor cell death to areas of innate and adaptive systemic antitumor immunity relevant to OPC.

**TABLE 1:** Major cell-death mechanisms and immunological outcomes relevant to immune escape and antitumour regulation in OPC.

Death modality/immune outcome	Core molecular mechanisms	Evidence context/level	Mechanisms of immune escape	Antitumour effects and therapeutic implications	References
Therapy-induced apoptosis with STING-associated immune activation (apoptosis–ICD interface)	The STING agonist dazostinag activates STING signalling within the tumour microenvironment, induces IFN-I signalling, and, in combination with chemotherapy, increases cleaved caspase-3 expression to promote tumour cell apoptosis	Human HNSCC; phase 0 intratumoral microdosing and spatial-profiling study; clinical mechanistic evidence, not OPSCC-specific	The HNSCC tumour microenvironment is characterized by immunosuppressive features, including immunosuppressive macrophage polarization and impaired immune surveillance; STING activation can partially reverse these immunosuppressive phenotypes	Dazostinag with chemotherapy induced type I IFN signalling, CXCL9-associated cytotoxic T-cell enrichment and increased apoptosis, supporting further clinical evaluation of STING activation combined with cytotoxic therapy; ICI efficacy was not directly tested	(Gregory et al., 2025)
Inflammasome activation (pyroptotic execution not directly demonstrated)	Increased expression of NLRP3, ASC, caspase-1, IL-1β and IL-18 supports inflammasome activation; gasdermin cleavage and membrane-pore formation were not assessed	Human OPSCC tissues; observational evidence for inflammasome activation; pyroptosis not directly demonstrated	Immune escape was not directly demonstrated; the immunological consequences of inflammasome activation in OPSCC remain uncertain	The findings support involvement of inflammasome signalling in OPSCC, but its antitumour function and therapeutic value require direct functional validation	(Takano et al., 2016)
Pyroptosis	DI@Lipo liposomes co-deliver cisplatin (DDP) and indocyanine green (ICG), activate the NLRP3 inflammasome through the synergistic effects of phototherapy and chemotherapy, promote GSDMD activation, and trigger pyroptosis; pyroptosis further leads to the release of DAMPs and inflammatory cytokines	HNSCC cell and murine models; preclinical experimental evidence	Some HNSCC tumours exhibit a low immune response state; DI@Lipo-induced pyroptosis can release DAMPs, promote the generation of immunogenic death signals, remodel a low-immune-response tumour state, and enhance immune cell recruitment	DI@Lipo-induced pyroptosis promotes DC maturation and increases cytotoxic T-cell infiltration; in HNSCC mouse models, it not only inhibits primary tumour growth but also induces an abscopal antitumour effect, providing a translational strategy for combining pyroptosis with immunotherapy	(Zhang et al., 2026a)
Pyroptosis	After the CXCR4-targeted nanotoxins T22-PE24-H6 and T22-DITOX-H6 enter HNSCC cells with high CXCR4 expression, they induce caspase-3 activation, which further cleaves GSDME to generate the GSDME N-terminal fragment, thereby forming membrane pores and leading to GSDME-dependent pyroptosis	HNSCC experimental models; preclinical evidence	In conventional HNSCC treatment, cancer cells develop resistance to chemotherapy and radiotherapy by evading apoptosis; high CXCR4 expression further promotes treatment resistance and tumour progression; this study overcomes part of the resistance to cell death by switching the mode of cell death from apoptosis to caspase-3/GSDME-mediated pyroptosis	After CXCR4-targeted nanotoxins induce tumour cell pyroptosis, they can increase the number of tumour-associated macrophages (TAMs), promote immune cell recruitment, and enhance antitumour effects	(Rioja-Blanco et al., 2022)
Ferroptosis	As an epigenetic regulator of ferroptosis, CRB2 inhibits the interaction between LSD1(E8A) and USP7, promotes LSD1(E8A) degradation, increases H4K20me2 levels in the SLC7A11 promoter region, suppresses SLC7A11 transcription, reduces the SLC7A11-GPX4 antioxidant defence pathway, and promotes lipid peroxidation and ferroptosis	HNSCC experimental models; preclinical mechanistic evidence	This study did not directly investigate immune escape-related molecules such as PD-1/PD-L1, Treg cells, and NK cells; restoring ferroptosis sensitivity reduced tumour-cell resistance to cell death, but its relevance to immunotherapy requires direct validation	CRB2 overexpression enhances the sensitivity of HNSCC cells to the ferroptosis inducers erastin and RSL3 and inhibits tumour cell growth; CRB2 combined with the ferroptosis inducer IKE can further enhance the *in vivo* antitumour effect, suggesting that the CRB2/ferroptosis axis has therapeutic potential	(Zhang et al., 2026c)
Ferroptosis	Ferroptosis-related genes and radiotherapy responses jointly participate in the regulation of HNSCC; the study found that radiotherapy can induce increased expression of the pro-ferroptotic molecules VDAC1 and TFRC while reducing the expression of the anti-ferroptotic factors GPX4, SLC7A11, and FTH1, suggesting that radiotherapy promotes tumour cell death by regulating the ferroptosis pathway	HNSCC bioinformatic analysis with limited cell validation; correlative/predictive evidence	Different ferroptosis–radiotherapy score groups were associated with differences in immune-cell infiltration and immunophenoscores; the F&Rs is a molecular classifier and should not be interpreted as a direct measure of active ferroptosis	Ferroptosis- and radiotherapy-related molecular features may support prognostic or treatment-response stratification; their predictive utility for immunotherapy and their value as therapeutic targets require prospective validation	(Fan et al., 2025)
Ferroptosis	As a negative regulator of ferroptosis, BASP1 affects the immune microenvironment of HNSCC by inhibiting immunogenic ferroptosis; BASP1 knockdown can induce ferroptosis, characterized by increased LDH release, elevated ROS levels, enhanced lipid peroxidation, and reduced GSH synthesis	HNSCC bioinformatic cohorts plus *in vitro* assays; mixed correlative/preclinical evidence	High BASP1 expression is associated with reduced CD8^+^ T-cell infiltration, T-cell dysfunction, and enhanced immune escape; tumours with high BASP1 expression exhibit an immunosuppressive microenvironment	BASP1 silencing experimentally induced ferroptosis in HNSCC cells, whereas associations with immune infiltration and immunotherapy response were primarily computational or correlative; whether BASP1 targeting improves ICI efficacy in HNSCC requires direct validation	(Pan et al., 2023)
Ferroptosis	DDR1 forms a DDR1/14–3–3/Akt complex through 14–3–3 mediation, activates Akt/mTORC1/SREBP1/SCD1 signalling, promotes MUFA biosynthesis, and inhibits ferroptosis; DDR1 inhibition reduces SREBP1/SCD1 expression, increases lipid peroxidation, Fe^2+^ accumulation, and ROS levels, and promotes ferroptosis	HNSCC experimental models; preclinical mechanistic evidence	High DDR1 expression is associated with decreased expression of CD8B, GZMB, IFNG, and IL12B and reduced T-cell infiltration, promoting immune escape and treatment resistance in HNSCC	DDR1 inhibition combined with CIR induces ferroptosis-mediated ICD, increases CRT and HSP90 exposure, promotes CD8^+^ T-cell infiltration and cytotoxic function, and thereby enhances antitumour immunity; the DDR1 inhibitor 7rh combined with CIR further promotes ferroptosis and enhances CD8^+^ T-cell infiltration, providing a strategy for precision treatment of HNSCC	(Hu et al., 2025)
PANoptosis (including pyroptosis, apoptosis, and necroptosis)	The engineered oncolytic virus VSV-S inserts the SMAC/DIABLO gene into the VSV genome to increase SMAC expression; VSV-S enhances pyroptosis by activating caspase-1/GSDMD signalling, while also promoting caspase-dependent apoptosis and MLKL phosphorylation-associated necroptosis, ultimately inducing PANoptosis	HNSCC cell lines and orthotopic tongue tumour model; preclinical evidence, not direct OPSCC evidence	HNSCC tumours can activate the PD-1/PD-L1 immunosuppressive axis through PD-L1 expression, reducing the antitumour function of T cells; VSV-S infection can reduce PD-L1 levels in tumour cells and alleviate T-cell exhaustion	VSV-S-induced PANoptosis promotes CD8^+^ T-cell infiltration into tumours and enhances cytotoxic T-cell function; combined PD-1 blockade further increases CD8^+^ T-cell numbers, killing capacity, and antitumour effects, providing a strategy for combining PANoptosis with immune checkpoint therapy	(Chen et al., 2025b)
Necroptosis	CGKRK-modified lipid nanoparticles deliver shikonin (SHK) and induce necroptosis in HNSCC cells; C-SNPs promote the phosphorylation of RIP1 and MLKL, induce DAMP release, and promote ICD	HNSCC experimental models; preclinical evidence	Poor immune infiltration and limited responsiveness to ICB can occur in subsets of HNSCC; in the reported model, C-SNPs induced necroptosis and inflammatory signalling, with evidence of enhanced immune recruitment	C-SNPs induce DAMP release, promote DC recruitment and maturation, and enhance CD8^+^ T-cell-mediated antitumour immunity; Mn^2+^-modified C-SMNPs further enhance cGAS-STING signalling and improve the therapeutic effect of PD-1 blockade	(Yu et al., 2025)
ICD	A Golgi-targeted protoporphyrin-RVRR photosensitive platform (GT-RG) was constructed, using the furin-cleavable property of the RVRR peptide sequence to achieve Golgi localization and generate ROS through photodynamic action, thereby inducing ICD	Head and neck carcinoma/OSCC-related models; preclinical evidence, not OPSCC-specific	Tumour-associated macrophages (TAMs) participate in the formation of an immunosuppressive tumour microenvironment and limit antitumour immune responses; this platform improves the immune environment by capturing CSFR^+^ TAMs and reprogramming them	GT-RGEV@CSFRi induces ICD, promotes TAM-associated immune microenvironment remodelling, and enhances the antitumour therapeutic effect in combination with anti-PD-1	(Zhou et al., 2024b)
ICD	Hypofractionated radiotherapy induces ICD in HNSCC cells and promotes the release of DAMPs, including CRT exposure, HMGB1 release, and ATP release; these signals promote DC activation and tumour antigen presentation, enhancing adaptive immune responses	HNSCC cell and mouse models with supportive human correlative data; preclinical/translational evidence	HNSCC forms immune escape through low tumour antigen release, insufficient immune cell infiltration, and accumulation of immunosuppressive cells such as MDSCs and Tregs; radiotherapy-induced PD-L1 expression may also form negative feedback that suppresses T-cell function	Hypofractionated radiotherapy induces ICD, increases CRT, HMGB1, and ATP levels, and promotes the activation of CD8^+^ T cells and DCs; combined anti-PD-L1 therapy can enhance antitumour immune responses, improve treatment sensitivity, and establish immune memory	(Mao et al., 2023)

Evidence context/level distinguishes direct OPSCC, evidence from HNSCC-, OSCC-, and other tumour-derived evidence and differentiates clinical, preclinical experimental, and bioinformatic/predictive findings. ICD, is presented as a functional immunological outcome rather than a distinct cell-death execution pathway. HNSCC-derived findings are included when OPSCC-specific evidence is limited and should be interpreted as mechanistic context unless directly validated in OPSCC.

**FIGURE 4:**
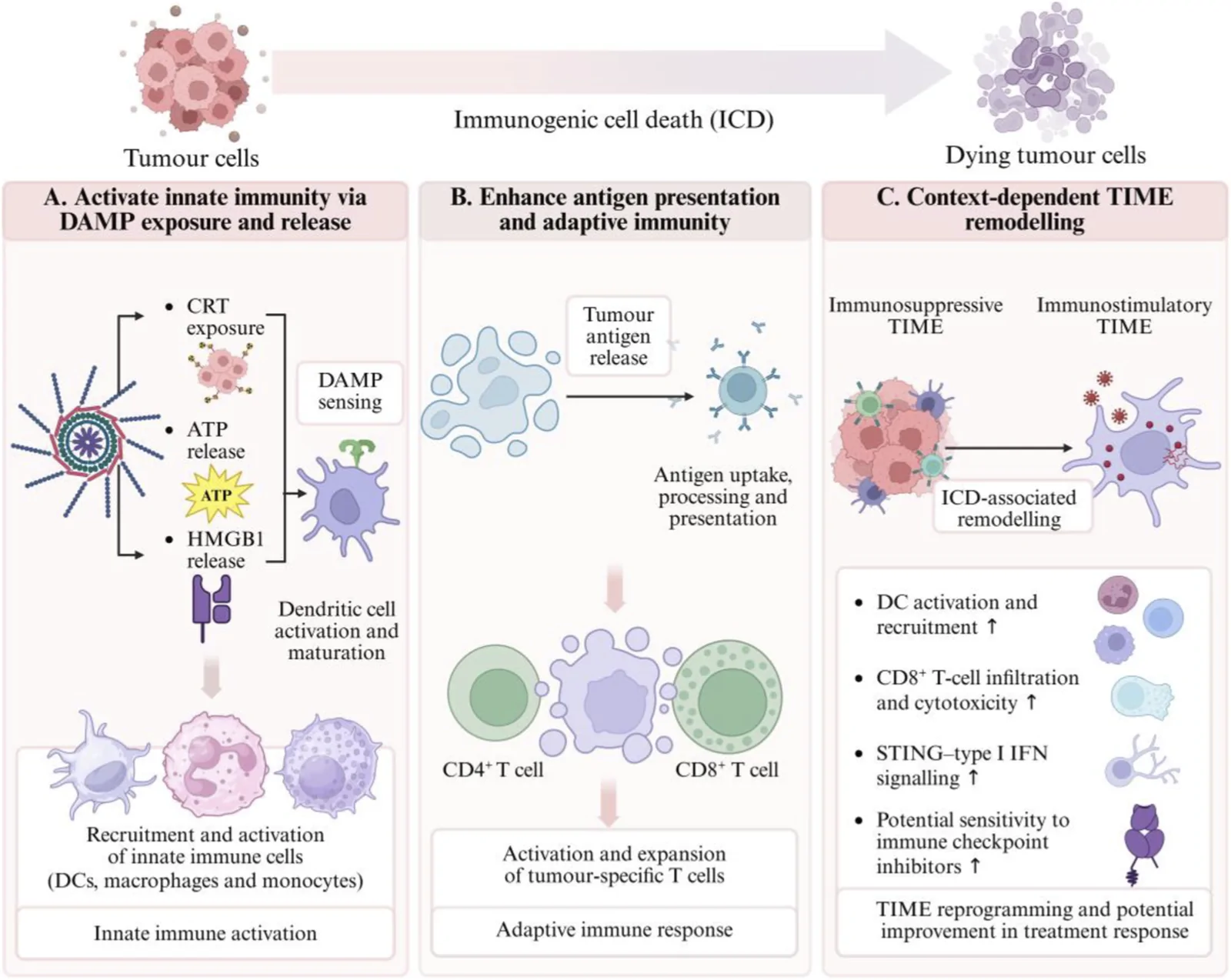
ICD-associated activation of innate and adaptive antitumour immunity relevant to OPSCC. This figure summarizes different ways in which informative signals induced in dying tumor cells can influence immune activation by linking to ICD. **(A)** Exposure of CRT and release of ATP and HMGB1 induce an innate immune response. These signals are sensed by innate immune cells, induce DC activation and maturation, and facilitate the recruitment of macrophages and monocytes to the TIME. **(B)** ICD facilitates the delivery of tumor-associated antigens: Tumor antigen release increases antigen availability, uptake and processing/presentation by APCs, which promotes activation/expansion of tumor-reactive CD4^+^ and CD8^+^ T cell adaptive immunity. **(C)** ICD-associated remodeling may redirect the TIME from an immunosuppressive state toward a more immunostimulatory state through increased DC recruitment, CD8^+^ T cell infiltration and cytotoxicity, STING–IFN-I signaling and potential sensitivity to ICIs. Where direct OPSCC evidence is less forthcoming, HNSCC-derived or other tumor evidence provides a mechanistic rationale.

## 6 Translational implications and therapeutic strategies

### 6.1 Combining PCD with immunotherapy

PCD eliminates tumor cells and modulates antitumor immunity by generating immunogenic changes, releasing immunostimulatory signals and reshaping the TIME. Generally, the efficacy of ICIs is limited in a proportion of patients by an immunosuppressive TIME, poor effector T cell infiltration and activation, and immune evasion. Immunoregulatory PCD might improve the immunogenicity of tumors and/or the antitumor immune response, consequently increasing sensitivity to ICIs (Yu et al., 2025).

PCD-induced immunogenicity is a major mechanism through which ICIs can be potentiated. PCD-based combination immunotherapy promotes immunogenic tumour-cell clearance and remodels the TIME, increasing sensitivity to immune-checkpoint blockade (Chen F. et al., 2025). Combining PCD induction with ICIs can also release immunostimulatory signals, restore T cell-mediated effector function and strengthen antitumour immunity (Ghosh et al., 2025). As the central immune programme linking PCD to immune activation, ICD increases tumour immunogenicity and the efficacy of ICIs by releasing stimulatory signals, enhancing antigen presentation and promoting T cell responses. Inducing immunoregulatory PCD together with checkpoint blockade may therefore help to overcome tumour immune tolerance.

Metabolism-associated PCD provides additional targets for combination immunotherapy, particularly through ferroptosis. This form of PCD is jointly controlled by metabolic networks and redox homeostasis and can alter the TIME through tumour–immune interactions as well as determining tumour-cell survival. Ferroptosis-associated remodeling of the tumor microenvironment increased response to immune checkpoint blockade (ICB) in prostate cancer models (Deng et al., 2024). In lung cancer models, inhibition of CPT1A induced ferroptosis and increased antitumor efficacy of immunotherapy (Ma et al., 2024). These observations provide mechanistic context for combination strategies of ferroptosis and immunotherapy, but relevance to OPSCC must be established. Manipulating the dynamic balance between metabolic state and PCD sensitivity may therefore refine immunotherapeutic strategies.

Beyond ferroptosis, several PCD modes may be productively combined with ICIs. GSDME-dependent pyroptosis promoted antitumor immunity and efficacy of anti-PD-1 therapy in colorectal cancer models (Gao et al., 2025). Mitochondrial apoptosis has been targeted to increase the sensitivity of tumor cells to NK-cell-mediated killing and the efficacy of NK-cell-based immunotherapy across experimental cancer models (Pan et al., 2022). Modulating PCD during ICI treatment can coordinate immune-cell activation with tumour-cell clearance and further strengthen antitumour immunity (Ghosh et al., 2025). In bladder cancer models, induction of cuproptosis combined with PD-L1 blockade enhanced antitumour immunity (Guo et al., 2023), providing cross-tumour mechanistic support for further investigation of this strategy in OPSCC. Each PCD mode nevertheless has distinct immunoregulatory properties, and therapeutic effects depend on the mode and conditions of induction, metabolic context and local immune environment. Precise control must therefore be tailored to the tumour context.

The combination of PCD and immunotherapy is not limited to clearance of tumor cells, but also converts death signals into drivers of immune regulation and provides a potential mechanism for ICI sensitization. Nevertheless, current mechanistic evidence in HNSCC is mainly preclinical and requires clinical definition for optimal timing, dosing and safety boundaries of combinations inducing PCD (Yu et al., 2025; Hsieh et al., 2024). To translate PCD-mediated immune sensitization to OPC care, mechanistic and clinical studies will need to be more closely integrated.

### 6.2 Targeting PCD to enhance radiotherapy and chemotherapy

Radiotherapy and chemotherapy are still at the core of OPC treatment, but tumor cell death escape, metabolic adaptation and dysregulated immune control can contribute to resistance. PCD orchestrates the response to therapeutic stress and can modulate treatment response by altering death susceptibility, affecting ICD induction and reshaping the local immune environment (Grottker et al., 2023; Song et al., 2024).

Targeting the execution machinery of PCD can restore tumour-cell susceptibility to death and improve radiotherapy and chemotherapy. Cell-death and stress-response pathways can modulate sensitivity or resistance to radiotherapy and chemotherapy, as demonstrated in studies of OPC and related HNSCC models (Grottker et al., 2023; Qiao et al., 2017; Cui et al., 2026). Mitochondrial dysfunction-dependent PCD networks regulate therapeutic stress adaptation. Moreover, mitochondrial targeting overcomes chemoresistance via coactivation of apoptosis and ferroptosis in doxorubicin-resistant breast cancer models (Hao et al., 2025); however, the relevance of these mechanisms to OPSCC remains unknown. Targeting inhibitor of apoptosis proteins (IAPs) can promote caspase-dependent cell-death signalling and enhance the efficacy of cisplatin-based chemoradiotherapy in locally advanced HNSCC, with exploratory evidence of benefit in HPV-negative OPC (Sun et al., 2020; Yungan et al., 2023), underscoring the need to interpret PCD-based treatment sensitization according to HPV context rather than generalizing across OPSCC subgroups. Manipulating key PCD nodes and death thresholds may therefore increase therapy-induced tumour-cell clearance and improve radiotherapy and chemotherapy responses.

PCD-induced immunogenicity can add an immune-regulatory component to radiotherapy and chemotherapy. Targeting immunogenic PCD promotes the release of DAMPs, including ATP and HMGB1, enhances dendritic-cell-mediated tumour recognition and T cell-mediated immunity, and increases tumour sensitivity to PD-1 blockade (Zhao et al., 2025). Pyroptosis mediated by caspase-3/GSDME can convert part of chemotherapy-induced apoptosis into inflammatory death with immune-activating properties, promoting immunostimulatory-signal release, tumour-cell clearance and treatment response (Wang et al., 2017). This conversion changes the mode of tumour-cell death and can amplify treatment-induced antitumour effects by recruiting immune cells and improving the local immune environment.

Metabolism-associated PCD offers another route to overcome resistance to radiotherapy and chemotherapy. Ferroptosis is closely regulated by redox homeostasis and antioxidant defence. In hepatocellular carcinoma models, SOCS2-mediated SLC7A11 degradation promoted ferroptosis and radiosensitization (Chen Q. et al., 2023), providing cross-tumour mechanistic context rather than direct evidence in OPSCC. Ferroptosis may also enhance multimodal treatment by altering the local immune microenvironment. Coordinating PCD pathways with conventional radiotherapy and chemotherapy could align tumour-cell death with immune activation and improve overall antitumour efficacy. Dynamic interactions among metabolic state, PCD susceptibility and treatment-induced damage may therefore provide a mechanistic basis for sensitization in OPC.

Manipulating PCD pathways can alter the response of tumors to radiotherapy or chemotherapy, either by altering the mode of cell death or by changing tumor cell susceptibility to killing. This sensitization differs based on death type, treatment stimulus and local microenvironment (Noh et al., 2025; Sarnella et al., 2022). Understanding how PCD programs interact with resistance sets the stage for the rational design of OPC treatment-sensitization strategies.

### 6.3 Precision immunotherapy for HPV-related OPC

HPV + OPSCC exhibits unique immune features driven by HPV and, in a permissive immune context, may be associated with TIME remodeling and PCD-linked immune regulation that may influence responses to cancer immunotherapy. While HPV + OPSCC often exhibits substantial immune infiltration, its clinical response to immunotherapy is heterogeneous; checkpoint-associated restraint and PCD-linked immune regulation can still shape clinical benefit (Chung et al., 2023; Cha et al., 2024).

Immunotherapy for HPV-related OPSCC requires integrated assessment of viral-antigen features and tumour immune state. HPV-encoded proteins such as E6/E7 serve as virus-derived tumour-associated antigens that continuously shape tumour–immune interactions and the immune microenvironment. HPV + OPSCC exhibits a distinct immune microenvironment characterized by enhanced immune-cell infiltration and increased immune-checkpoint expression, and clinical responses to PD-1 blockade support further exploration of rational combination immunotherapy strategies (Ferris et al., 2021). Integrating molecular subtype, TIME state and PCD-related features may further refine immune activation and treatment precision (Wang X. et al., 2021). Patient stratification by virological features and immune ecology is therefore a prerequisite for individualized immunotherapy in HPV + OPSCC.

PCD modulation may offer a route to sensitize HPV-related OPSCC to immunotherapy. Immunogenic PCD can improve tumour recognition and therapeutic response by releasing DAMPs, enhancing antigen presentation and recruiting immune cells. Similarly, HNSCC and murine tumors with PD-L1 expression mediated by ferroptotic stress were even more responsive to anti-PD-L1 treatment following priming with a ferroptosis inducer; however, the relationship between ferroptosis-associated signatures and immune activation was stronger in HPV-negative than in HPV-positive HNSCC (Chung et al., 2023). PD-L1 expression in tonsillar squamous cell carcinoma was correlated with HPV status and features of epithelial-mesenchymal transition (EMT), but exhibited considerable intratumoral heterogeneity. Therefore, the joint assessment of PD-L1 expression, HPV status and markers associated with EMT may facilitate defining clinically different subsets of patients (Kwon et al., 2018). PCD state may thus coordinate viral immune features, TIME remodelling and immunotherapy outcome as well as determining the mode of tumour-cell death.

Multi-omics and immune-biomarker analyses are advancing PCD-informed individualized immunotherapy for HPV-related OPSCC. Single-cell multi-omics can resolve the functional states and clonal characteristics of immune-cell subsets and immunoregulatory molecules in OPSCC, identify cellular factors associated with immune-checkpoint response, and thereby inform treatment optimization (Cheng et al., 2020). These and other studies performed in OPSCC support the potential of adopting HPV/p16 status, PD-L1 and/or other molecular or circulating biomarkers for risk stratification and treatment response, but prospective validation is needed (Chen et al., 2026). Mechanistic studies of HNSCC suggest that HPV-encoded circE7 can impede CD8^+^ T-cell infiltration and activity via the ACC1-LGALS9/galectin-9-associated pathway, supporting the rationale for simultaneous PD-1 and TIM-3 blockade (Ge et al., 2024). Integrating TIME regulation, PCD induction and immune activation may further reduce immunosuppression and improve treatment efficacy (Wu et al., 2023).

Utilizing HPV-associated immune features, a spectrum of immune checkpoints and PCD-mediated immune remodeling-based approaches may support molecularly directed precision immunotherapy for HPV-related OPSCC and potentially improve clinical benefit. The relationships between PCD modes, viral features and immune ecology are not fully understood, and the biomarkers of current ICI therapies often do not capture response heterogeneity. Resolving PCD-dependent immune networks and further refinement of precision immunotherapy in HPV-related OPSCC will be reliant on multidimensional biological profiling (Chung et al., 2023; Cha et al., 2024). Table 2 summarizes PCD-oriented translational strategies and biomarker-based approaches with potential relevance to OPC, along with evidence contexts and applicable settings. These therapeutic principles position the targeting of PCD as a conduit connecting tumor cell death and immune activation. As illustrated by Figure 5, the addition of PCD modulation to immunotherapy could further enhance precision therapeutic approaches for OPC and be combined with conventional treatments and HPV-informed biomarker stratification.

**TABLE 2:** PCD-oriented translational strategies and biomarker-based approaches with potential relevance to OPC.

Therapeutic strategy	Targeted cell death type/key node	Combination treatment	Evidence context/applicable setting	Major translational value	References
Aurora kinase A-targeted inhibition enhances immunotherapy	Aurora A inhibition → DNA damage → caspase-3/PARP-mediated apoptosis; caspase-3/GSDME-mediated ICD-related pyroptosis; CRT exposure and DAMP release promote immune activation	Alisertib combined with PD-1 or CTLA-4 ICB	HPV-driven cancer models, including HPV-positive HNSCC; preclinical experimental evidence relevant to HPV-associated OPSCC	By inducing ICD, reducing MDSCs and enhancing CD8^+^ T-cell function, Aurora A inhibition increased sensitivity to ICI therapy in preclinical HPV-driven cancer models	(Ghosh et al., 2025)
Aurora kinase A-targeted inhibition enhances sensitivity to immunotherapy	Aurora A inhibition → G2/M arrest, DNA damage, and mitotic catastrophe in the context of Rb deficiency; induction of apoptosis and ICD, promoting cGAS-STING-mediated immune activation	Alisertib combined with PD-1 ICB (pembrolizumab)	Immunotherapy- and platinum-resistant HPV-positive HNSCC; phase I/II clinical evidence	Preclinical studies support Aurora A inhibition-induced ICD and immune sensitization; clinically, alisertib plus pembrolizumab produced prolonged stable disease in a subset of immunotherapy-resistant HPV-positive HNSCC patients, although no objective responses were observed	(Johnson et al., 2025)
IAP inhibition combined with TRAILR2/DR5 death receptor activation restores apoptosis sensitivity	Extrinsic apoptosis; TRAIL/TRAILR2 (DR5)–FADD–caspase-8 axis and IAP/cIAP1/XIAP anti-apoptotic nodes	The SMAC mimetic birinapant combined with TRAIL and/or TRAILR2/DR5 agonistic antibodies	HPV-positive HNSCC cell models; preclinical experimental evidence	Relieving IAP-mediated inhibition of death signalling and enhancing TRAILR2-dependent caspase-8-mediated apoptosis provide a preclinical rationale for death-pathway-targeted combinations in HPV-positive HNSCC, with validation required in OPSCC	(An et al., 2021)
HPV E6 inhibition-mediated radiosensitization therapy	Apoptosis; HPV E6–p53 regulatory axis; caspase-8 activation and the apoptotic execution pathway mediated by caspase-3/PARP cleavage	GA-OH combined with photon radiotherapy or proton radiotherapy	HPV-positive and HPV-negative HNSCC cell models, with stronger radiosensitization in HPV-positive cells; *in vitro* preclinical evidence relevant to HPV-associated OPSCC	Provides preclinical support for HPV E6 inhibition as a radiosensitization strategy; clinical applicability to radiotherapy de-escalation in HPV-positive OPSCC remains to be established	(Chitsike et al., 2023)
Glutamine metabolism inhibition combined with CD47 blockade enhances radiotherapy-induced ferroptosis	Ferroptosis; SLC1A5/glutamine metabolism–IRF1–transferrin receptor–Fe^2+^ axis; CD47-mediated escape from macrophage phagocytosis	Glutamine inhibition combined with radiotherapy and CD47 blockade	HNSCC experimental models with radiotherapy resistance; preclinical mechanistic evidence	By enhancing IRF1-dependent ferroptosis and relieving CD47-mediated inhibition of macrophage phagocytosis, tumour regression is promoted and the tumour microenvironment is improved, providing a basis for combining ferroptosis with immune regulation to enhance radiotherapy	(Song et al., 2024)
Statin-induced ferroptosis combined with radiotherapy sensitization strategy	Ferroptosis; NCOA4/DMT1 promotes iron metabolism and ferroptosis activation, while GPX4/FTH1 mediates anti-ferroptotic defence	Atorvastatin or simvastatin combined with ionizing radiation (IR)	HPV-negative HNSCC patient cohorts plus *in vitro* and *in vivo* models; prognostic and preclinical radiosensitization evidence	Promotes Fe^2+^ accumulation and lipid peroxidation, induces ferroptosis, enhances radiosensitivity, and inhibits tumour growth	(Noh et al., 2025)
ALA-PDT induces ferroptosis and remodels the antitumour immune microenvironment	Ferroptosis; the STEAP3/p53/SLC7A11 regulatory axis promotes lipid peroxidation and ferroptosis while driving M1-like macrophage polarization	ALA-PDT-mediated ferroptosis and macrophage immune regulation strategy	HPV-associated cellular models, including HPV16-positive SiHa cells and macrophage experiments; preclinical cross-disease evidence, not direct OPSCC evidence	Provides cross-disease mechanistic context for HPV-associated ferroptosis–macrophage interactions; relevance to HPV-positive OPSCC requires direct validation	(Xu et al., 2026)
Targeting the Chemerin/NLRP3/GSDMD-N pyroptosis pathway as a potential strategy in OSCC	Pyroptosis; Chemerin promotes NLRP3 inflammasome activation, inducing caspase-1-mediated GSDMD-N formation and IL-1β release	Potential pyroptosis pathway inhibition strategies, including NLRP3 inhibition; combination approaches require further validation	Human OSCC tissues plus *in vitro* and nude-mouse models; OSCC-specific preclinical evidence, requiring validation in OPSCC	The Chemerin/NLRP3/GSDMD-N axis is associated with pyroptosis, EMT, migration, invasion, cell proliferation and tumour growth, providing a potential target for further therapeutic investigation in OSCC	(Zhang et al., 2026d)
Triptolide (TPL)-induced GSDME-mediated pyroptosis therapeutic strategy	Pyroptosis; TPL inhibits the c-Myc/HK-II axis, promotes BAD/BAX-caspase-3 activation, and induces GSDME cleavage and GSDME-N-mediated membrane pore formation	TPL combined with the ferroptosis inducer erastin enhances tumour killing by inhibiting the NRF2/SLC7A11 axis and increasing ROS accumulation	Head and neck cancer cell and xenograft models; preclinical experimental evidence, not OPSCC-specific	Induces GSDME-dependent pyroptosis, promotes IL-1β and LDH release, and inhibits tumour cell survival; combination with erastin further enhances antitumour effects *in vitro* and *in vivo* and has the potential to activate antitumour immunity	(Cai et al., 2021)
Cisplatin-induced RIP1/RIP3/MLKL-mediated necroptosis therapeutic strategy	Necroptosis; the NF-κB/TNF-α autocrine axis activates the RIP1/RIP3/MLKL signalling pathway	Cisplatin monotherapy induces necroptosis and can be further explored in combination with necroptosis-regulating strategies	Human HNSCC cell lines HN4 and SCC4; *in vitro* preclinical evidence	Cisplatin activated NF-κB/TNF-α signalling and RIP1/RIP3/MLKL-dependent necroptotic signalling in HNSCC cells, providing mechanistic evidence for cisplatin-associated necroptosis; therapeutic benefit from additional necroptosis-targeting combinations remains unvalidated	(Wang et al., 2024)
Candidate therapeutic strategy associated with the cuproptosis-related lncRNA JPX regulatory network	Cuproptosis-related lncRNA regulatory network involving the JPX/miR-193b-3p/PLAU axis; the study did not directly demonstrate cuproptotic cell-death execution	Axitinib and palbociclib were identified as candidate agents through expression-associated drug-sensitivity and predicted IC50 analyses; direct pharmacological inhibition of JPX or PLAU was not experimentally demonstrated	HNSCC multi-omics, single-cell and *in vitro* experimental study with computational drug-sensitivity prediction; not OPSCC-specific	The JPX/miR-193b-3p/PLAU axis provides a mechanistic and hypothesis-generating therapeutic framework; the proposed axitinib–palbociclib combination requires direct pharmacological, preclinical and OPSCC-specific validation	(Sun et al., 2024)
Risk stratification and treatment-response prediction based on CRGs	CRG signature including FDX1, LIAS, LIPT1, DLD, DLAT, PDHA1, PDHB, MTF1, GLS and CDKN2A; this signature does not directly measure active cuproptosis	Bioinformatically predicted ICB response and chemotherapy sensitivity; no therapeutic intervention was directly tested	TCGA-based HNSCC cohort; bioinformatic prognostic and predictive evidence	The CRG-based model was associated with prognosis, immune-cell infiltration and predicted immunotherapy response, supporting potential biomarker-based stratification; it does not establish active cuproptosis or efficacy of cuproptosis-targeted therapy	(Ding et al., 2022)

Evidence context distinguishes direct clinical evidence from preclinical experimental and bioinformatic/predictive findings. HNSCC-, OSCC-, HPV-associated non-OPSCC, and other disease models are included as mechanistic or translational context when OPSCC-specific evidence is unavailable. Predicted drug sensitivity or molecular signatures should not be interpreted as direct pharmacological target validation or evidence of active execution of the corresponding cell-death pathway.

**FIGURE 5:**
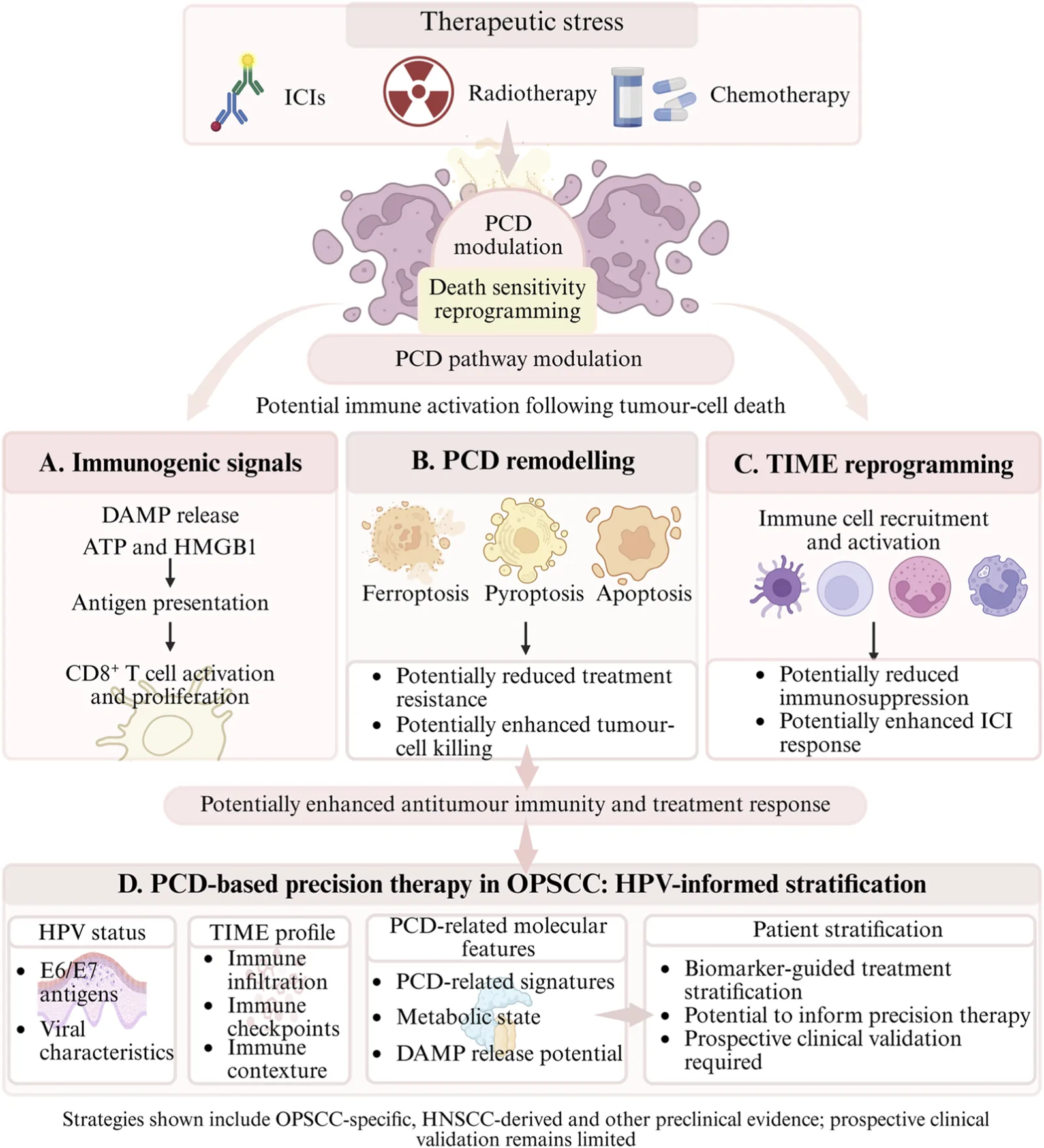
Therapeutic modulation of PCD to enhance antitumour immunity and support precision therapy in OPSCC. Schematic representation of the translational significance of PCD modulation in OPSCC therapy. Therapeutic modalities, such as ICIs or radio- and chemotherapy, can alter PCD pathways and reprogram the sensitivity of cancer cells to death, which has the potential for immune activation upon cell death. **(A)** The immunogenic signals generated during PCD, such as the release of ATP and HMGB1, may augment antigen presentation and stimulate CD8^+^ T cell activation and proliferation. **(B)** PCD with tuned pathways (ferroptosis, pyroptosis and apoptosis) may diminish treatment resistance and bolster tumor cell killing. **(C)** TIME reprogramming may recruit and activate immune cells, reduce immunosuppression while potentially further augmenting ICI response. **(D)** Based on HPV status, viral antigen features and TIME profiles, along with immune checkpoints, PCD-related molecular features, metabolic state, DAMP release potential and integrative multi-omics, biomarker-based treatment stratification is desirable for OPSCC; prospective validation is still warranted. Strategies are depicted, with evidence from OPSCC-specific as well as HNSCC-derived and other preclinical studies, whereas direct clinical validation is still scarce.

## 7 Discussion

PCD links tumour-cell fate to immune regulation. Distinct PCD modes can alter the TIME and immunotherapy response, providing a framework for understanding immune escape and treatment heterogeneity in OPC (Wang S. et al., 2023; Zhang J. et al., 2024). This Review has examined the continuum of immunological consequences associated with different cell death modalities, which range from relative immunological silence to ICD, treated as a functionally relevant outcome rather than an independent execution pathway, and summarized the potentially diverse signalling effects of ferroptosis, pyroptosis, necroptosis and cuproptosis on TIME remodeling and treatment response. This Review has also examined potential links between PCD programmes, immune escape and immunotherapy sensitivity in the viral and immune context of HPV-related OPC.

Despite broad interest in PCD as a regulator of tumour immunity, important limitations remain in OPC. A major limitation is that many mechanistic studies of PCD regulation are derived from HNSCC rather than OPSCC-specific models. Although these findings provide valuable biological insights due to shared squamous carcinoma characteristics, future studies using OPSCC-specific models and patient cohorts are required to validate these mechanisms. Ferroptosis, pyroptosis and other PCD modes contribute to HNSCC progression and immune-microenvironmental regulation, but their specific roles in OPC, interactions among death modes and dynamics under therapeutic pressure remain poorly characterized (Yu et al., 2022; Li M. et al., 2022). Molecular signatures and computational models alone should not be taken as direct evidence of cell-death execution, although most current evidence comes from transcriptomic analyses, *in vitro* models and animal studies; hence only limited direct evidence currently supports *bona fide* ACD in OPSCC. While spatial multi-omics, single-cell sequencing and longitudinal monitoring have partially interrogated the true tumor ecology in patients, few studies have integrated these approaches to examine the relationship between a PCD network and clinical response. HPV-positive and HPV-negative OPSCC also differ considerably in virus-related molecular characteristics, immune infiltration and treatment response (Liu X. et al., 2022), yet the specific links between PCD and immune escape in each HPV context are not well defined. Current PCD-based strategies largely target individual death pathways; approaches that coordinate multiple modes remain limited. Achieving tumour selectivity while establishing durable antitumour immunity is therefore a central translational challenge.

Future work should resolve the complex PCD-dependent immune-regulatory network in OPC at several levels. Single-cell sequencing integrated with multi-omics can map interactions among tumour, immune and stromal cells, define the functions of distinct cellular niches and identify how PCD-related molecular features contribute to immune-microenvironmental heterogeneity (Zhang et al., 2023; Kürten et al., 2021). Longitudinal analysis of PCD switching under therapeutic pressure should determine how radiotherapy, chemotherapy and immunotherapy alter death modes and, in turn, antigen presentation, immune-cell recruitment and checkpoint expression. Patient-stratification systems that integrate HPV status, virus-associated molecular features, TIME classification and PCD-related markers could clarify potential associations between PCD features and immunotherapy response and support stratification in OPC (Kim et al., 2020; Xu S-M. et al., 2022). Evaluating PCD inducers together with ICIs, radiotherapy, chemotherapy and delivery systems should also help to optimize combinations while limiting toxicity.

The integration of discoveries in tumor immunology and precision medicine over the past decade has expanded the understanding of PCD-linked pathways from mere mechanisms for cell death to regulators that integrate cancer biology and immune response, as well as dictate treatment sensitivity. Defining the dynamic interrelationship of PCD modes, virus-related immune characteristics and TIME in OPC may clarify key mechanisms of immune evasion, facilitating a transition toward mechanism-based precision intervention away from empirical combinations. Multidimensional biomarker systems capturing PCD-related features may help to better explain the interpatient variability of immunotherapy response and thus advance precision immunotherapy in OPC (Chung et al., 2023; Cha et al., 2024).

## Glossary

ACD: autophagy-dependent cell death
APCs: antigen-presenting cells
ATP: adenosine triphosphate
CIR: carbon ion radiotherapy
CRG(s): cuproptosis-related gene(s)
CRT: calreticulin
CTLA-4: cytotoxic T-lymphocyte-associated protein 4
CTLs: cytotoxic T lymphocytes
DAMPs: damage-associated molecular patterns
DCs: dendritic cells
EMT: epithelial-mesenchymal transition
GPX4: glutathione peroxidase 4
GSDMD: gasdermin D
GSDME: gasdermin E
GSH: glutathione
HMGB1: high mobility group box 1
HNSCC: head and neck squamous cell carcinoma
HPV: human papillomavirus
IAPs: inhibitor of apoptosis proteins
ICB: immune checkpoint blockade
ICD: immunogenic cell death
ICI(s): immune checkpoint inhibitor(s)
IFN-I: type I interferon
IRF1: interferon regulatory factor 1
LAG-3: lymphocyte activation gene 3
lncRNA: long non-coding RNA
M-MDSCs: monocytic myeloid-derived suppressor cells
MDSCs: myeloid-derived suppressor cells
MHC-I: major histocompatibility complex class I
mTORC1: mechanistic target of rapamycin complex 1
MUFA: monounsaturated fatty acid
NK cells: natural killer cells
NLRP3: NLR family pyrin domain containing 3
OPC(s): oropharyngeal cancer(s)
OPSCC: oropharyngeal squamous cell carcinoma
OSCC: oral squamous cell carcinoma
PCD: programmed cell death
PD-1: programmed cell death protein 1
PD-L1: programmed death-ligand 1
PDT: photodynamic therapy
PRRs: pattern recognition receptors
RCD: regulated cell death
ROS: reactive oxygen species
SLC7A11: solute carrier family 7 member 11
STING: stimulator of interferon genes
TAMs: tumour-associated macrophages
TCR: T cell receptor
TIGIT: T cell immunoreceptor with Ig and ITIM domains
TILs: tumour-infiltrating lymphocytes
TIME: tumour immune microenvironment
TIM-3: T-cell immunoglobulin and mucin domain-containing protein 3
TPL: triptolide
Tregs: regulatory T cells
Trm: tissue-resident memory T cells
xCT: cystine/glutamate antiporter
